# Identification of Candidate Olfactory Genes in *Scolytus schevyrewi* Based on Transcriptomic Analysis

**DOI:** 10.3389/fphys.2021.717698

**Published:** 2021-10-04

**Authors:** Xiaofeng Zhu, Bingqiang Xu, Zhenjie Qin, Abudukyoum Kader, Bo Song, Haoyu Chen, Yang Liu, Wei Liu

**Affiliations:** ^1^Key Laboratory of Integrated Pest Management on Crops in Northwestern Oasis, Ministry of Agriculture and Rural Affairs, Institute of Plant Protection, Xinjiang Academy of Agricultural Sciences, Urumqi, China; ^2^State Key Laboratory for Biology of Plant Diseases and Insect Pests, Institute of Plant Protection, Chinese Academy of Agricultural Sciences, Beijing, China; ^3^Guangdong Laboratory for Lingnan Modern Agriculture (Shenzhen Branch), Genome Analysis Laboratory of the Ministry of Agriculture and Rural Affairs, Agricultural Genomics Institute at Shenzhen, Chinese Academy of Agricultural Sciences, Shenzhen, China

**Keywords:** *Scolytus schevyrewi*, transcriptome, olfactory genes, expression analysis, antennae

## Abstract

The bark beetle, *Scolytus schevyrewi (S. schevyrewi)*, is an economically important pest in China that causes serious damage to the fruit industry, particularly, in Xinjiang Province. Chemical signals play an important role in the behavior of most insects, accordingly, ecofriendly traps can be used to monitor and control the target pests in agriculture. In order to lay a foundation for future research on chemical communication mechanisms at the molecular level, we generate antennal transcriptome databases for male and female *S. schevyrewi* using RNA sequencing (RNA-seq) analysis. By assembling and analyzing the adult male and female antennal transcriptomes, we identified 47 odorant receptors (ORs), 22 ionotropic receptors (IRs), 22 odorant-binding proteins (OBPs), and 11 chemosensory proteins (CSPs). Furthermore, expression levels of all the candidate OBPs and CSPs were validated in different tissues of male and female adults by semiquantitative reverse transcription PCR (RT-PCR). *ScosOBP2* and *ScosOBP18* were highly expressed in female antennae. *ScosCSP2, ScosCSP3*, and *ScosCSP5* were specifically expressed in the antennae of both males and females. These results provide new potential molecular targets to inform and improve future management strategies of *S. schevyrewi*.

## Introduction

Olfaction serves to detect environmental chemical information necessary for insect behavior such as finding food sources, mates, and oviposition sites (Hanson, 1999; Clyne et al., 2000). Insects have a sophisticated olfactory system that begins with the reception of odorants at the peripheral chemosensory system. Insect olfaction is dependent on olfactory receptor neurons (ORNs) in sensilla (Leal, 2013) distributed mainly in antennae and also in maxillary palps or labial palps (Stoker et al., 1990). The research of molecular mechanisms of olfactory reception in insects has predominantly been in the model organism *Drosophila melanogaster*. These studies have shown diverse olfactory genes encoding proteins, such as odorant receptors (ORs), ionotropic receptors (IRs), odorant-binding proteins (OBPs), and chemosensory proteins (CSPs), involved in different chemical signal transduction processes (Benton et al., 2009; Wilson, 2013; Xiao et al., 2019).

Odorant receptors play a critical role in recognizing thousands of odorant molecules in the insect olfactory system. Insect ORs were first identified in *Drosophila* which has the characteristic feature of a seven-transmembrane domain (TMD) structure that is unrelated to the ORs in vertebrates (Clyne et al., 1999; Benton et al., 2006). Every ORN can express a single or two OR genes (Vosshall and Hanson, 2011). Specificity of OR relies on the ligand-banding ORs (Dobritsa et al., 2003; Elmore et al., 2003; Hallem et al., 2004), while Orco functions as an obligatory chaperon for the Orco-OR complex (Larsson et al., 2004; Benton et al., 2006; Stengl, 2017).

Evolved from the ionotropic glutamate receptor superfamily, IRs have been shown to be involved in odor reception. They are expressed in the sensory neurons that respond to many distinct odors, such as acids, amines, and other chemicals that cannot be recognized by ORs (Benton et al., 2006). Aside from olfaction, IRs serve various functions, such as cool sensation (Ni et al., 2015), hygrosensation (Knecht et al., 2016), circadian clock (Chen et al., 2015), and detection of carbon dioxide (CO_2_) (Breugel et al., 2018).

In addition to ORs and IRs, other multigene families encode proteins that also play critical roles in olfaction. OBPs are small soluble proteins secreted in the sensillar lymph. They are characterized by an N-terminal signal peptide sequence and a set of six conserved cysteine residues that form three disulfide bridges (Pelosi et al., 2005, 2006). Studies of defective mutants and wild-type counterparts of OBP76a (also known as LUSH) in *Drosophila* have shown that this protein has a key role in the perception of alcohol and 11-cis vaccenyl acetate (Kim et al., 1998; Xu et al., 2005; Gomez-Diaz et al., 2013). OBPs have also been reported as a pheromone-binding protein in *Lepidoptera* (Jing et al., 2019). Some OBPs operate similar to LUSH in response to pheromones. *In vivo* studies have shown that OBPs significantly affect pheromone perception in moths. Knocking out these OBPs significantly reduced electrophysiological responses to pheromones in several species, such as *Helicoverpa armigera* (Ye et al., 2017), *Spodoptera litura* (Liu et al., 2012; Zhu et al., 2016a), and *Chilio suppressalis* (Dong et al., 2017).

Chemosensory proteins are also small soluble proteins but are shorter in amino acid sequence length than that of OBPs, and CSPs share the same structure of having four conserved cysteines forming two disulfide bridges (Pelosi et al., 2005, 2006; Honson et al., 2015). As semiochemical carriers, some CSPs are involved in chemodetection (Pelosi et al., 2018; Li et al., 2021) because CSPs are abundant in the lymph of chemosensory hairs (Angeli et al., 1999; Jacquin-Joly et al., 2001; Monteforti et al., 2002; Sun et al., 2014). Some of them already have been reported to function such as OBPs, e.g., CSP3 of the honeybee, which specially binds some components of brood pheromone (Briand et al., 2002).

Bark beetles (Coleoptera; Curculionidae; Scolytinae) feed on woods and several of them pose serious threats to forestry, e.g., *Ips typographus* (Wermelinger, 2004)*, Dendroctonus ponderosae* (Andersson et al., 2013). Since their host-finding relies on chemical communication, e.g., aggregation behavior based on male-produced pheromone (Schlyter et al., 1987), pheromone-based technique could be used for the detection and control of this pest. In order to develop this technique efficiently, one way is to exploit olfactory genes that are critical for successful mate and host finding. Transcriptomic and genomics studies have been performed for searching olfactory genes in bark beetles (Andersson et al., 2013, 2019; Mitchell et al., 2019), and functional studies were limited to only seven ORs (Hou et al., 2021; Yuvaraj et al., 2021). *Scolytus schevyrewi* (*S. schevyrewi*) (Cleoptera: Scolylidae) is one of the most destructive insect pests of fruit trees in China. It has a wide host range and has been reported to attack several families of trees in Xinjiang province (Li et al., 1995). Several studies have focused on the identification and field bioassay of chemical attractants in the bark beetle (Fan et al., 2014). In order to provide a molecular basis for gene targets for putative chemical lures of this pest, we performed Illumina Hiseq 2000 sequencing of the transcriptome of adult male and female antennae samples.

## Materials and Methods

### Insect Rearing and Tissue Collection

*Scolytus schevyrewi* larvae were reared on the branches of their host plants (Armeniaca vulgaris) collected from Baren County, Xinjiang province, China (39.0°N, 75.8°E) and maintained in the lab under the following conditions of 26.5°C, a cycle of 14-h light:10-h dark, and 65% relative humidity. Pupae were placed on a branch and the emerged adults were collected every day. Two-day-old adults were used to collect male and female antennae, heads (without antennae), thorax, abdomen, legs, and wings using the fine-tip forceps, immediately frozen in liquid nitrogen and stored at−80°C until RNA isolation.

### RNA Extraction

Total RNA from different tissues of *S. schevyrewi* was obtained using TRIzol reagent (Invitrogen, Carlsbad, California, USA) following the instruction of manufacturer. The total RNA from each pair of antennae, legs, and wings were separately obtained from each adult, totaling 300 males and 300 females. Heads (without antennae), thoraxes, and abdomens were separately collected from 20 to 30 adult males and 20 to 30 females. Total RNA was dissolved in RNase-free water, and RNA integrity was verified by gel electrophoresis. RNA concentration and purity were determined on the Nanodrop ND-2000 spectrophotometer (NanoDrop products, Wilmington, DE, USA).

### cDNA Library Construction and Sequencing

A total of 1 μg of total RNA of each sample of male and female antennae were used to construct two separate cDNA libraries, one for each sex. Paired-end reads of 100 bp were sequenced using the Illumina HiSeq 2000 platform to obtain library-sequencing information at Beijing Genome Institute (Shenzhen, China). The detailed protocols for cDNA library construction and sequencing applied have been described in the previous studies (Cao et al., 2014; Zhang et al., 2015). The raw data were uploaded to the NCBI SRA database (Accession: PRJNA732801, https://www.ncbi.nlm.nih.gov/sra).

### Assembly

Low-quality reads were filtered out, low-quality nucleotides at each end were trimmed, and 3′ adaptors and poly-A/T were removed from the raw reads to generate the clean reads. Subsequently, the clean reads were used to form a *de novo* assembly using the Trinity platform (v2.1.0) with default parameters (Grabherr et al., 2011). The Trinity outputs were then clustered by TIGR gene indices clustering tools (TGICL) to generate the final unigene dataset (Pertea et al., 2003).

### Identification of Olfactory Genes

Unigenes were annotated using blastx against NCBI nonredundant (nr) sequences with *e* < 1e^−5^. The blast results were then imported into the Blast2Go (version 3.1) with default parameters (Conesa et al., 2005). OR, IR, OBP, and CSP genes of the candidates were selected according to the nr sequence annotation results in the remote server from the lab. All candidate olfactory genes were manually checked using the blastx program against the nr sequence database. The open-reading frames (ORFs) of the putative olfactory genes were predicted using the ExPASy (Expert Protein Analysis System) translate tool (https://web.expasy.org/translate/). The TMDs of ORs and IRs were predicted using TMHMM server version 2.0 (http://www.cbs.dtu.dk/services/TMHMM). Putative N-terminal signal peptides of OBPs and CSPs were predicted using the SignalP 4.0 server (http://www.cbs.dtu.dk/services/SignalP-4.0/) with default parameters.

### Phylogenetic Analysis

Olfactory genes from *S. schevyrewi, Ips typographus, Dendroctonus ponderosae* (Andersson et al., 2013), and *Holotrichia parallela* (Yi et al., 2018) were selected for the phylogenetic analysis. Sequence information was listed in Supplementary Table 2. Amino acid sequences were aligned by MAFFT (https://www.ebi.ac.uk/Tools/msa/mafft/). Phylogenetic trees of olfactory genes were constructed using RAxML version 8 with the Jones-Taylor-Thornton amino acid substitution model. Node support was assessed using a bootstrap method based on 1,000 replicates. The trees were visualized, and color-coded in FigTree 1.4.3. For ORs, the tree was rooted in the Orco lineage.

### Expression Analysis of the Candidate OBPs and CSPs by Semiquantitative Reverse Transcription PCR (RT-PCR)

Reverse transcription-PCR was performed to verify the expression patterns of OBPs and CSPs of *S. schevyrewi*. Total RNA from male and female antennae, heads (without antennae), thoraxes, legs, abdomens, and wings were used to synthesize cDNA by RevertAid First Strand cDNA Synthesis Kit (Thermo Scientific, Waltham, MA, USA). Gene-specific primers were designed using Primer 5 and synthesized by Sangon Biotech Co., Ltd. (Shanghai, China) (Supplementary Table 1). PCR was performed with the Veriti Thermal Cycler (Applied Biosystems, Carlsbad, CA, USA) under the following conditions: 95°C for 3 min, 25 cycles at 95°C for 30 s, 55°C for 30 s, 72°C for 30 s, and 72°C for 10 min. PCR amplification products were run on a 2% agarose gel. Because it is difficult to acquire massive amounts of RNA from antennae samples of *S. schevyrewi*, only two technical repeats were performed for each gene. Uncropped gel images were uploaded as supplements (Supplementary Figure 2).

## Results

### Transcriptome Assembly

The transcriptomes of male and female *S*. *schevyrewi* antennae were separately sequenced by the Illumina HiSeq 2000 platform. Then after filtering, 26,804,894 and 29,176,485 clean reads with 98.60 and 98.55% Q20 scores were generated for male and female samples, respectively. The clean reads were assembled subsequently and generated 40,666 and 36,216 unigenes, respectively. After merging and clustering, a final transcript dataset was revealed with 34,098 unigenes consisting of 14,071 clusters and 20,027 distinct singletons. The dataset was 46.7~57.4 Mb in size and with unigenes having a mean length of 1,684 bp and N50 of 3,179 bp.

### Gene Identification and Functional Annotation

The functional annotations of the unigenes were performed mainly based on the blastx results against the nr sequence database. We matched 22,815 (66.9%) unigenes to known proteins by blastx. Among those annotated genes, 16,725 (73.3%) unigenes showed strong homology (*e*-values lower than 1e^−45^), while 6,090 (26.7%) unigenes showed poor matches with *e*-values between 1e^−15^ and 1e^−5^. The similarity analysis showed that 11,514 (50.5%) unigenes had more than 60% similarity with known proteins. Most of the annotated unigenes were matched to *Tribolium castaneum* (67.3%), followed by *D. ponderosae* (13.7%) and others species (19.0%).

Gene ontology (GO) annotations of the entire set of unigenes were performed using the Blast2GO pipeline based on the blastx searches against nr sequences. A total of 12,720 unigenes were assigned various GO terms. In the molecular function category, genes involved in the binding activity and catalytic activity were most abundant. In the cellular component category, genes involved in cell, cell part, macromolecular, membrane, organelle, and organelle part were enriched. In the biological process category, genes involved in the cellular process, metabolic process and single-organism process were the most represented.

### Identification of Candidate Odorant Receptors

The candidate ORs were identified by keyword search of the blastx annotations. We identified 47 putative OR genes. Thirteen of them were full-length putative OR genes ranging from 1,100 to 1,400 bp with complete ORFs and 5 to 7 TMDs, which are characteristics of typical insect ORs. This includes the full-length ScosOrco gene encoding 488 amino acids. Seven of the predicted incomplete ORs were shorter in length and contained a deduced protein longer than 300 amino acids. Four of the predicted incomplete ORs were even shorter than 200 amino acids.

**Figure 1:**
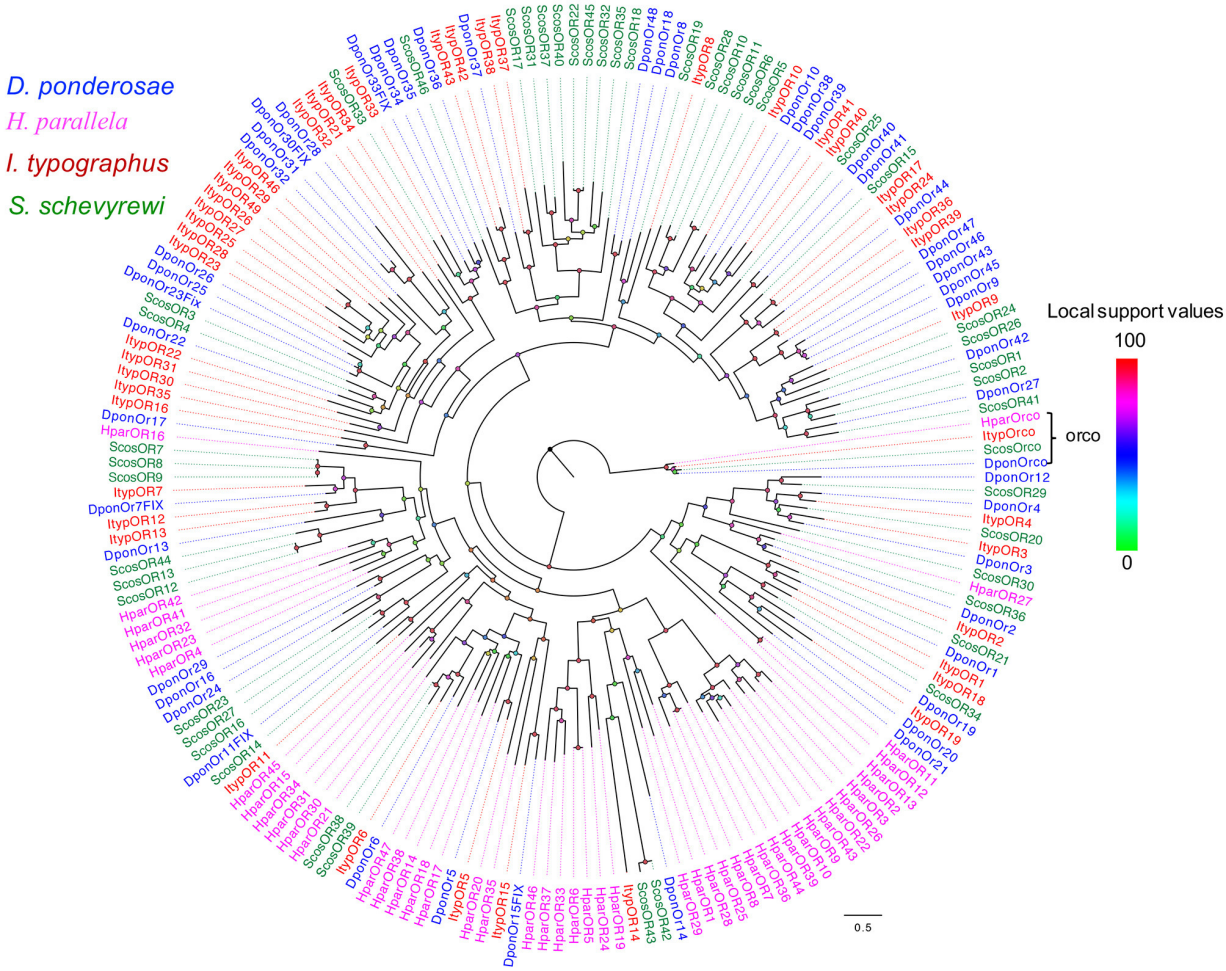
Phylogenetic tree of candidate ScosORs with known Coleoptera odorant receptors sequences. Dpon, *D. ponderosae*; Hpar, *H. parallela*; Ityp, *I. typographus*; Scos, *S. schevyrewi*.

The blastx results indicated that the identities of the most predicted ORs shared with known insect ORs were very low, ranging from 24 to 49%. Nine predicted ORs (ScosOR1, ScosOR27, ScosOR7, ScosOR38, ScosOR39, ScosOR2, ScosOR8, ScosOR9, and ScosOR34) had greater identity (52–62%) with the OR from *D. ponderosae*. ScosOrco had 88% identity with the Orco from *Rhynchophorus ferrugineus*. Phylogenetic analysis was performed with ORs from *D. ponderosae, I. typographus, H. parallela*, and *S. schevyrewi* (Figure 1). A branch for Orco was identified in the phylogenetic tree. Two expanded branches in this species relative to others in the comparison were also identified. One branch consisted of ScosOR5, ScosOR6, ScosOR10, ScosOR11, ScosOR25, and ScosOR28 and the other consisted of ScosOR17, ScosOR18, Scos22, Scos31, Scos32, Scos37, Scos40, and Scos45. Most of the branches in the tree were supported by high-local support values and few branches were not reliable.

Information on unigene reference, length, and best blastx hit of all 47 ORs are listed in Table 1.

**Table 1:** Unigenes of candidate odorant receptors.

**Unigene reference**	**Gene name**	**Length (bp)**	**ORF(aa)**	**Blastx best hit (Reference/Name/Species)**	***E*-value**	**Full length**	**TMD**
CL839.Contig1_All	ScosOR1	735	244	XP_019765879.1 PREDICTED: odorant receptor 94a-like [Dendroctonus ponderosae]	1.00E-129	No	3
CL839.Contig2_All	ScosOR2	1,143	380	XP_019765879.1 PREDICTED: odorant receptor 94a-like [Dendroctonus ponderosae]	2.00E-144	Yes	6
CL1001.Contig1_All	ScosOR3	1,251	416	XP_019754377.1 PREDICTED: putative odorant receptor 92a [Dendroctonus ponderosae]	5.00E-92	No	6
CL1001.Contig2_All	ScosOR4	1,365	454	XP_019762540.1 PREDICTED: odorant receptor 49b-like [Dendroctonus ponderosae]	4.00E-92	No	7
CL1025.Contig1_All	ScosOR5	1,173	390	XP_019765587.1 PREDICTED: odorant receptor Or2-like [Dendroctonus ponderosae]	5.00E-57	No	5
CL1025.Contig2_All	ScosOR6	996	331	XP_019765587.1 PREDICTED: odorant receptor Or2-like [Dendroctonus ponderosae]	3.00E-45	No	4
CL1127.Contig1_All	ScosOR7	1,131	376	XP_019762033.1 PREDICTED: odorant receptor 4-like isoform X2 [Dendroctonus ponderosae]	4.00E-133	Yes	5
CL1127.Contig2_All	ScosOR8	1,155	384	XP_019762033.1 PREDICTED: odorant receptor 4-like isoform X2 [Dendroctonus ponderosae]	2.00E-144	Yes	6
CL1127.Contig4_All	ScosOR9	1,149	382	XP_019762033.1 PREDICTED: odorant receptor 4-like isoform X2 [Dendroctonus ponderosae]	5.00E-144	Yes	6
CL1283.Contig1_All	ScosOR10	1,188	395	XP_019755291.1 PREDICTED: odorant receptor 47b-like [Dendroctonus ponderosae]	4.00E-63	No	4
CL1283.Contig2_All	ScosOR11	774	257	AKK25156.1 odorant receptor 15 [Dendroctonus ponderosae]	3.00E-64	No	4
CL1562.Contig1_All	ScosOR12	891	296	XP_018564120.1 PREDICTED: odorant receptor 85b-like [Anoplophora glabripennis]	6.00E-19	No	6
CL1562.Contig2_All	ScosOR13	1,156	384	XP_018564120.1 PREDICTED: odorant receptor 85b-like [Anoplophora glabripennis]	2.00E-33	No	7
CL2075.Contig3_All	ScosOR14	1,455	484	ALR72569.1 odorant receptor OR26 [Colaphellus bowringi]	2.00E-57	No	7
CL2243.Contig2_All	ScosOR15	1,191	396	XP_019754447.1 PREDICTED: odorant receptor 49b-like [Dendroctonus ponderosae]	9.00E-59	No	7
CL2311.Contig1_All	ScosOR16	1,137	378	XP_019770928.1 PREDICTED: odorant receptor 94a-like [Dendroctonus ponderosae]	6.00E-127	Yes	7
CL2593.Contig1_All	ScosOR17	1,173	390	AKK25156.1 odorant receptor 15 [Dendroctonus ponderosae]	1.00E-20	No	5
CL2643.Contig2_All	ScosOR18	1,176	391	XP_019874691.1 PREDICTED: odorant receptor 67c-like [Aethina tumida]	3.00E-27	No	6
CL2759.Contig1_All	ScosOR19	377	125	XP_019768086.1 PREDICTED: odorant receptor Or2-like [Dendroctonus ponderosae]	1.00E-29	No	2
CL2885.Contig1_All	ScosOR20	1,206	401	XP_019771895.1 PREDICTED: odorant receptor 67c-like, partial [Dendroctonus ponderosae]	3.00E-106	Yes	7
CL3312.Contig1_All	ScosOR21	1,176	391	XP_018567969.1 PREDICTED: odorant receptor Or2-like [Anoplophora glabripennis]	4.00E-54	Yes	6
Unigene7_All	ScosOR22	1,179	392	XP_019772797.1 PREDICTED: odorant receptor Or2-like [Dendroctonus ponderosae]	3.00E-18	No	7
Unigene529_All	ScosOR23	367	122	AKK25157.1 odorant receptor 17, partial [Dendroctonus ponderosae]	6.00E-15	No	2
Unigene2373_All	ScosOR24	1,167	388	XP_019761187.1 PREDICTED: odorant receptor Or2-like [Dendroctonus ponderosae]	8.00E-75	Yes	4
Unigene2403_All	ScosOR25	1,179	424	XP_019753281.1 PREDICTED: odorant receptor 47b-like [Dendroctonus ponderosae]	5.00E-92	Yes	6
Unigene3424_All	ScosOR26	1,170	389	XP_019761187.1 PREDICTED: odorant receptor Or2-like [Dendroctonus ponderosae]	4.00E-199	Yes	6
Unigene3466_All	ScosOR27	1,134	377	XP_019770928.1 PREDICTED: odorant receptor 94a-like [Dendroctonus ponderosae]	1.00E-138	No	7
Unigene3624_All	ScosOR28	1,179	392	AKK25156.1 odorant receptor 15 [Dendroctonus ponderosae]	9.00E-72	No	5
Unigene3644_All	ScosOR29	1,176	391	XP_019756949.1 PREDICTED: odorant receptor 67c-like isoform X2 [Dendroctonus ponderosae]	3.00E-119	Yes	7
Unigene4009_All	ScosOR30	1,185	394	XP_018571501.1 PREDICTED: odorant receptor 67c-like [Anoplophora glabripennis]	1.00E-49	No	6
Unigene6079_All	ScosOR31	1,122	373	XP_019765855.1 PREDICTED: odorant receptor 49b-like [Dendroctonus ponderosae]	1.00E-23	No	3
Unigene7306_All	ScosOR32	1,188	395	XP_019765855.1 PREDICTED: odorant receptor 49b-like [Dendroctonus ponderosae]	1.00E-26	No	6
Unigene8744_All	ScosOR33	372	123	XP_019771464.1 PREDICTED: odorant receptor 46a-like isoform X3 [Dendroctonus	3.00E-31	No	0
Unigene9796_All	ScosOR34	1,160	385	XP_019755672.1 PREDICTED: odorant receptor 4 [Dendroctonus ponderosae]	7.00E-133	Yes	6
Unigene10776_All	ScosOR35	1,158	385	XP_019759347.1 PREDICTED: odorant receptor 30a-like [Dendroctonus ponderosae]	8.00E-24	No	6
Unigene12156_All	ScosOrco	1,467	488	AOO35283.1 olfactory co-receptor [Rhynchophorus ferrugineus]	0.00E+00	Yes	7
Unigene12163_All	ScosOR36	1,184	393	XP_015836240.1 PREDICTED: odorant receptor 49b [Tribolium castaneum]	1.00E-114	No	6
Unigene12204_All	ScosOR37	1,134	378	XP_019874691.1 PREDICTED: odorant receptor 67c-like [Aethina tumida]	2.00E-24	No	5
CL90.Contig6_All	ScosOR38	1,236	411	XP_019768012.1 PREDICTED: odorant receptor 83a-like isoform X2 [Dendroctonus ponderosae]	2.00E-170	No	6
CL90.Contig22_All	ScosOR39	927	308	XP_019768012.1 PREDICTED: odorant receptor 83a-like isoform X2 [Dendroctonus]	7.00E-82	No	2
CL90.Contig22_All	ScosOR39	927	308	XP_019768012.1 PREDICTED: odorant receptor 83a-like isoform X2 [Dendroctonus]	7.00E-82	No	2
Unigene17267_All	ScosOR40	1,041	346	AKK25154.1 odorant receptor 7, partial [Dendroctonus ponderosae]	9.00E-08	No	2
Unigene18514_All	ScosOR41	370	123	XP_019765855.1 PREDICTED: odorant receptor 49b-like [Dendroctonus ponderosae]	1.00E-37	No	1
CL110.Contig4_All	ScosOR42	900	299	EFA01416.1 odorant receptor 283 [Tribolium castaneum]	1.00E-06	No	4
CL110.Contig8_All	ScosOR43	825	273	EFA01423.1 odorant receptor 293 [Tribolium castaneum]	4.60E+00	No	4
CL548.Contig1_All	ScosOR44	1,161	386	XP_015837918.1 PREDICTED: putative odorant receptor 85d [Tribolium castaneum]	7.00E-26	No	7
CL584.Contig4_All	ScosOR45	1,179	392	XP_019772797.1 PREDICTED: odorant receptor Or2-like [Dendroctonus ponderosae]	2.00E-18	No	8
CL733.Contig1_All	ScosOR46	1,176	391	XP_019765855.1 PREDICTED: odorant receptor 49b-like [Dendroctonus ponderosae]	6.00E-25	No	4

### Identification of Candidate Ionotropic Receptors

Bioinformatics analysis identified 22 putative IRs in the *S. schevyrewi* transcriptome. Only ScosIR76b was a full-length sequence with 555 amino acids and five TMDs; the other IRs were incomplete due to the lack of the 5′ or 3′ terminus.

**Figure 2:**
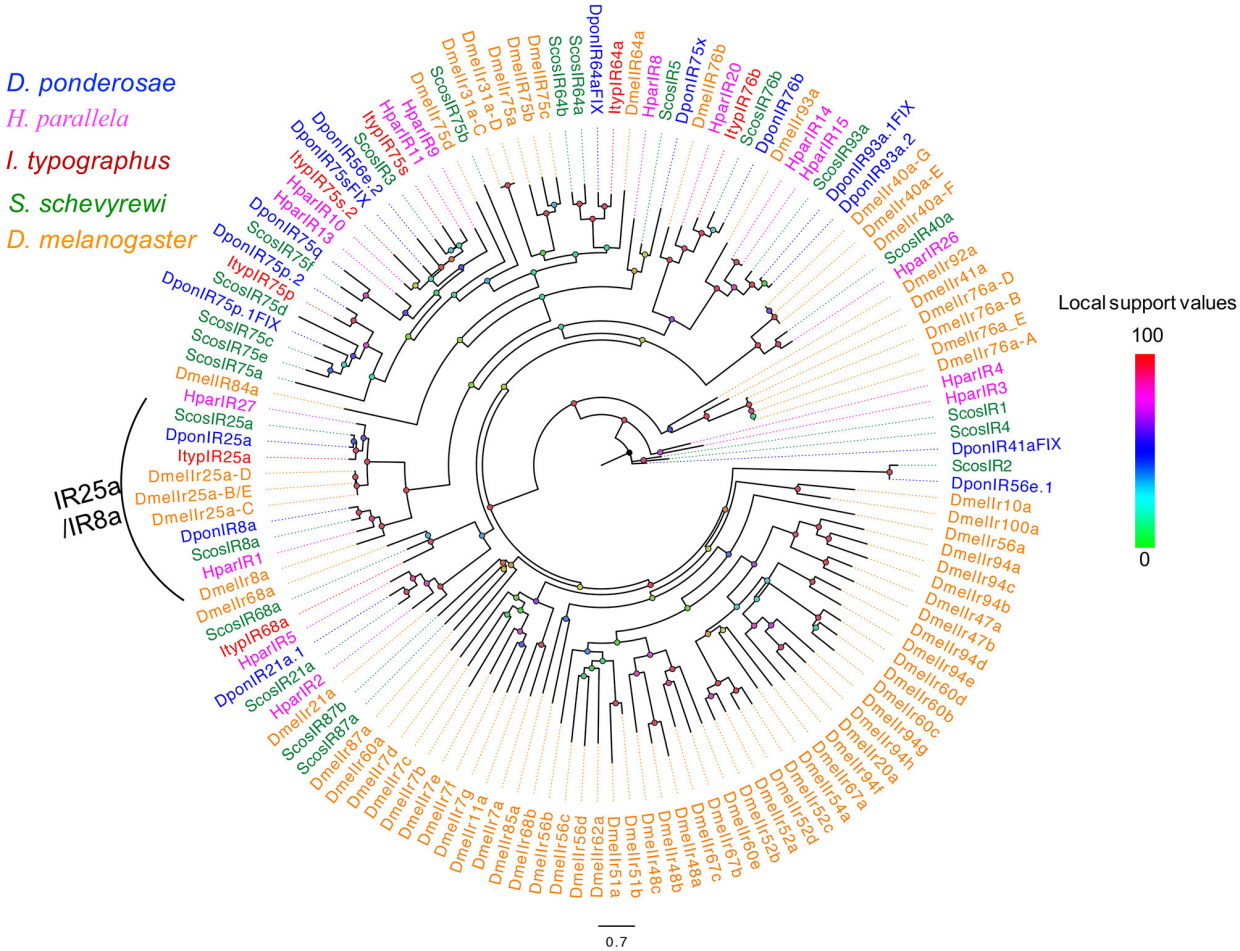
Phylogenetic tree of candidate ScosIRs with ionotropic receptor sequences from other insects. Tcas, *T. castaneum*; Dpon, *D. ponderosae*; Hpar, *H. parallela*; Ityp, *I. typographus*; Dmel, *D. melanogaster*; Scos, *S. schevyrewi*.

The blastx results showed that more than half of the predicted IRs (ScosIR1, ScosIR75a, ScosIR75b, ScosIR75c, ScosIR75d, ScosIR75e, ScosIR75f, ScosIR3, ScosIR4, ScosIR64a, ScosIR64b, and ScosIR87b) shared low identity (24–49%) with known insect IRs. A total of 10 predicted IRs (ScosIR68a, ScosIR93a, ScosIR76b, ScosIR2, ScosIR21a, ScosIR8a, ScosIR40a, ScosIR87a, ScosIR25a, and ScosIR5) had greater identity (54–95%) with known insect IRs, most of which were from *D. ponderosae*. Candidate genes with high identity (87%) to DponIR25a were deemed IR 25a homologs. A phylogenetic tree was constructed based on the IR sequences from *D. ponderosae, I. typographus, H. parallela*, and *S. schevyrewi* (Figure 2). ScosIR8a and ScosIR25a were identified as putative IR8a and IR25a homlogs due to the IR8a/IR25a branch.

Information on unigene reference, length, and best blastx hit of all 22 IRs are listed in Table 2.

**Table 2:** Unigene of candidate ionotropic receptors.

**Unigene reference**	**Gene name**	**Length (bp)**	**ORF(aa)**	**Blastx best hit (Reference/Name/Species)**	***E*-value**	**Full length**	**TMD**
Unigene10667_All	ScosIR1	1,497	498	XP_019865961.1 PREDICTED: LOW QUALITY PROTEIN: glutamate receptor 2-like [Aethina tumida]	4.00E-102	No	2
CL3060.Contig1_All	ScosIR2	927	308	ABD36125.1 glutamate receptor Gr2 [Bombyx mori]	2.00E-101	No	7
CL146.Contig15_All	ScosIR3	1,836	611	ALR72541.1 ionotropic receptor IR2 [Colaphellus bowringi]	5.00E-172	No	5
Unigene2622_All	ScosIR4	1,803	600	XP_019865961.1 PREDICTED: LOW QUALITY PROTEIN: glutamate receptor 2-like [Aethina tumida]	5.00E-108	No	4
Unigene14518_All	ScosIR5	319	106	XP_018563257.1 PREDICTED: glutamate receptor ionotropic, NMDA 2D-like [Anoplophora glabripennis]	5.00E-62	No	1
CL296.Contig4_All	ScosIR8a	2,649	882	XP_019770830.1 PREDICTED: ionotropic receptor 25a [Dendroctonus ponderosae]	0.00E+00	No	3
Unigene4531_All	ScosIR21a	2,370	789	XP_019753472.1 PREDICTED: ionotropic receptor 21a [Dendroctonus ponderosae]	0.00E+00	No	3
CL3341.Contig1_All	ScosIR25a	2,796	931	XP_019763174.1 PREDICTED: ionotropic receptor 25a [Dendroctonus ponderosae]	0	No	3
Unigene6808_All	ScosIR40a	539	179	XP_019764671.1 PREDICTED: glutamate receptor ionotropic, delta-2-like [Dendroctonus ponderosae]	2.00E-97	No	0
CL16.Contig22_All	ScosIR64a	1,860	619	XP_019770931.1 PREDICTED: glutamate receptor-like [Dendroctonus ponderosae]	5.00E-164	No	3
Unigene8833_All	ScosIR64b	1,863	620	XP_019770931.1 PREDICTED: glutamate receptor-like [Dendroctonus ponderosae]	7.00E-173	No	0
Unigene11157_All	ScosIR68a	357	118	XP_015839172.1 PREDICTED: glutamate receptor ionotropic, kainate 5 [Tribolium castaneum]	1.00E-27	No	1
Unigene5334_All	ScosIR75a	540	180	XP_021192228.1 glutamate receptor ionotropic, delta-1-like isoform X2 [Helicoverpa armigera]	1.00E-22	No	3
Unigene11324_All	ScosIR75b	1,533	510	XP_015836653.1 PREDICTED: glutamate receptor 2-like [Tribolium castaneum]	2.00E-72	No	4
CL266.Contig1_All	ScosIR75c	1,854	617	AKC58589.1 chemosensory ionotropic receptor 75q, partial [Anomala corpulenta]	3.00E-85	No	3
Unigene2679_All	ScosIR75d	390	129	APC94258.1 ionotropic receptor 2, partial [Pyrrhalta maculicollis]	6.00E-06	No	1
Unigene6978_All	ScosIR75e	1,893	630	XP_018572783.1 PREDICTED: glutamate receptor 1-like [Anoplophora glabripennis]	4.00E-77	No	3
CL1275.Contig2_All	ScosIR75f	1,626	541	XP_018572783.1 PREDICTED: glutamate receptor 1-like [Anoplophora glabripennis]	4.00E-92	No	3
Unigene11968_All	ScosIR76b	1,668	555	XP_019762016.1 PREDICTED: LOW QUALITY PROTEIN: glutamate receptor ionotropic, kainate 1-like [Dendroctonus ponderosae]	0.00E+00	Yes	5
Unigene2912_All	ScosIR87a	352	117	XP_017770100.1 PREDICTED: glutamate receptor ionotropic, NMDA 2B-like isoform [Nicrophorus vespilloides]	1.00E-101	No	0
Unigene1422_All	ScosIR87b	1,848	615	OWR45511.1 putative chemosensory ionotropic receptor IR68a [Danaus plexippus]	2.00E-054	No	5
Unigene874_All	ScosIR93a	2,598	865	XP_018576793.1 PREDICTED: glutamate receptor ionotropic, delta-1 isoform X2	0.00E+00	No	1

### Identification of Odorant-Binding Proteins

In the *S. schevyrewi* antennal transcriptome, 22 different sequences encoding putative OBPs were identified. More than half of them (ScosOBP2, ScosOBP4, ScosOBP8, ScosOBP9, ScosOBP10, ScosOBP11, ScosOBP14, ScosOBP15, ScosOBP16, ScosOBP19, ScosOBP20, and ScosOBP22) were identified as full-length sequences. The lengths of all full-length ScosOPBs ranged from 130 to 241 amino acids.

**Figure 3:**
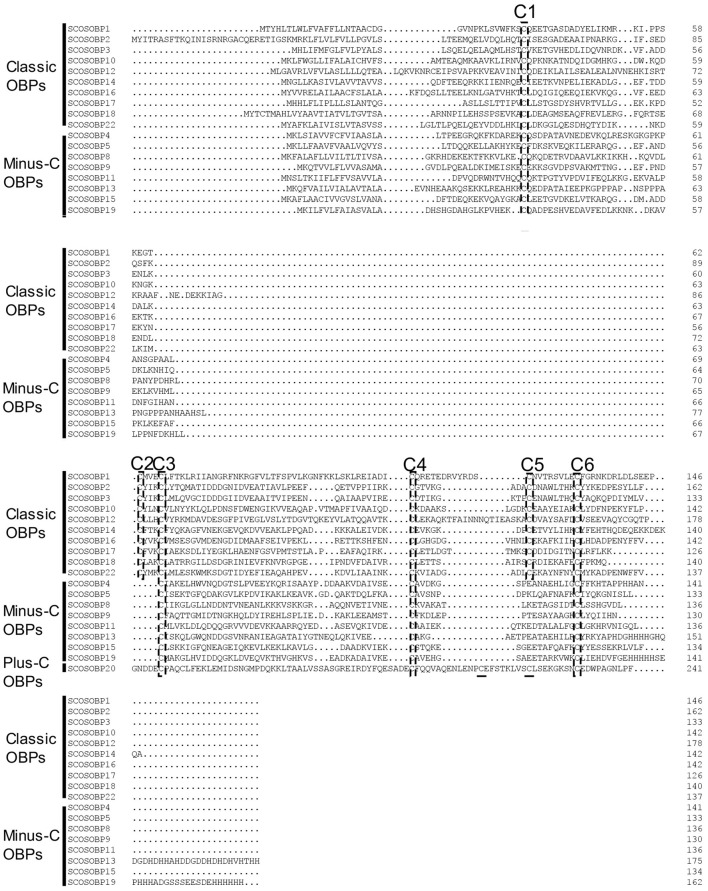
Sequence alignment of candidate ScosOBPs. The six conserved cysteine residues are marked as C1–C6.

**Figure 4:**
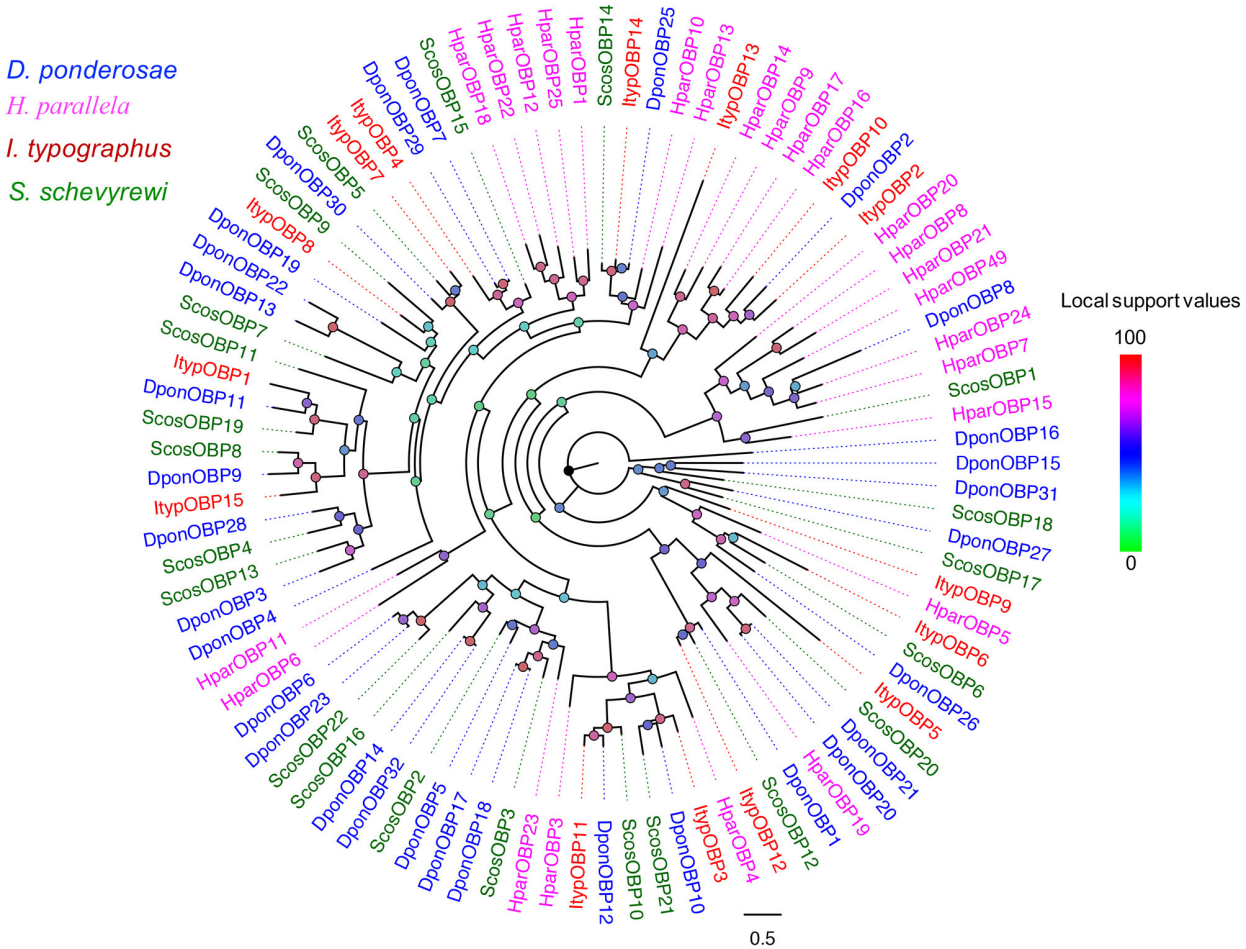
Phylogenetic tree of candidate ScosOBPs with known Coleoptera odorant-binding proteins sequences. Tcas, *T. castaneum*; Dpon, *D. ponderosae*; Hpar, *H. parallela*; Ityp, *I. typographus*; Scos, *S. schevyrewi*.

Nearly, half of the predicted OBPs (ScosOBP7, ScosOBP18, ScosOBP9, ScosOBP1, ScosOBP20, ScosOBP19, ScosOBP17, ScosOBP4, ScosOBP4, ScosOBP6, and ScosOBP8) shared relatively low identity with known insect OBPs (31–49%). A total of 12 predicted OBPs (ScosOBP13, ScosOBP15, ScosOBP11, ScosOBP16, ScosOBP21, ScosOBP5, ScosOBP2, ScosOBP3, ScosOBP22, ScosOBP12, ScosOBP10, and ScosOBP14) had greater identity (51–83%) with known OBPs, a majority of which were *D. ponderosae* OBPs. Sequence alignment showed that 10 OBPs (ScosOBP1, 2, 3, 10, 12, 14, 16, 17, 18, and 22) matched in amino acid sequence to the sequence structure of classic OBPs, and eight OBPs (ScosOBP4, 5, 8, 9, 11, 13, 15, and 19) matched the sequence structure of Minus-C OBPs (Figure 3). Other OBPs were not analyzed by sequence alignment due to their limited sequence lengths. In the phylogenetic analysis of OBPs of different coleopterans, ScosOBPs were found across various branches and generally formed small subgroups together with OBPs from other coleopterans (Figure 4). No species-specific branch was discovered.

Information on unigene reference, length, and best blastx hit of all 22 OBPs are listed in Table 3.

**Table 3:** Unigene of candidate odorant binding proteins.

**Unigene reference**	**Genename**	**Length (bp)**	**ORF(aa)**	**Blastx best hit**	***E*-value**	**Full length**	**Signal peptide**
CL1164.Contig1_All	ScosOBP1	441	146	ALR72497.1 odorant binding protein 9 [Colaphellus bowringi]	6.00E-24	No	Yes
CL2073.Contig1_All	ScosOBP2	489	162	AAQ96921.1 odorant-binding protein RpalOBP4', partial [Rhynchophorus palmarum]	6.00E-48	Yes	Yes
CL2693.Contig1_All	ScosOBP3	402	133	AKK25145.1 odorant binding protein 21 [Dendroctonus ponderosae]	4.00E-39	No	Yes
CL3244.Contig1_All	ScosOBP4	426	141	ALM64971.1 odorant binding protein 13 [Dendroctonus armandi]	2.00E-32	Yes	Yes
CL3634.Contig1_All	ScosOBP5	402	133	ALM64972.1 odorant binding protein 14 [Dendroctonus armandi]	9.00E-44	No	Yes
CL3848.Contig1_All	ScosOBP6	489	162	APG79364.1 pheromone binding protein 3 [Cyrtotrachelus buqueti]	1.00E-27	No	Yes
Unigene1743_All	ScosOBP7	358	119	ARU83754.1 odorant binding protein 3 [Anoplophora glabripennis]	9.00E-09	No	Yes
Unigene1760_All	ScosOBP8	411	136	AGI05185.1 odorant-binding protein 9 [Dendroctonus ponderosae]	2.00E-42	Yes	Yes
Unigene1792_All	ScosOBP9	393	130	AQY18983.1 odorant-binding protein [Galeruca daurica]	8.00E-18	Yes	Yes
Unigene4680_All	ScosOBP10	429	142	AKK25140.1 odorant binding protein 16 [Dendroctonus ponderosae]	3.00E-64	Yes	Yes
Unigene5401_All	ScosOBP11	411	136	AKK25135.1 odorant binding protein 9, partial [Dendroctonus ponderosae]	8.00E-44	Yes	Yes
Unigene6017_All	ScosOBP12	537	178	ARH65471.1 odorant binding protein 16 [Anoplophora glabripennis]	9.00E-69	No	Yes
Unigene7865_All	ScosOBP13	531	176	AMK48596.1 odorant-binding protein, partial [Rhynchophorus ferrugineus]	7.00E-24	No	Yes
Unigene9055_All	ScosOBP14	429	142	AHE13793.1 odorant binding protein [Lissorhoptrus oryzophilus]	2.00E-75	Yes	Yes
Unigene9643_All	ScosOBP15	405	134	AHE13799.1 odorant binding protein [Lissorhoptrus oryzophilus]	9.00E-40	Yes	Yes
Unigene9992_All	ScosOBP16	429	142	AHE13800.1 odorant binding protein, partial [Lissorhoptrus oryzophilus]	6.00E-50	Yes	Yes
Unigene10379_All	ScosOBP17	381	126	ALM64968.1 odorant binding protein 6 [Dendroctonus armandi]	7.00E-22	No	Yes
Unigene11422_All	ScosOBP18	423	140	ALM64970.1 odorant binding protein 8 [Dendroctonus armandi]	3.00E-09	No	Yes
Unigene11547_All	ScosOBP19	489	162	AGI05181.1 odorant-binding protein 11 [Dendroctonus ponderosae]	1.00E-18	Yes	Yes
Unigene12124_All	ScosOBP20	726	241	AGI05159.1 odorant-binding protein 21 [Dendroctonus ponderosae]	1.00E-43	Yes	Yes
Unigene17771_All	ScosOBP21	242	80	ANE37553.1 odorant binding protein 9 [Rhynchophorus ferrugineus]	1.00E-24	No	Yes
CL142.Contig2_All	ScosOBP22	414	137	AFI45061.1 odorant-binding protein [Dendroctonus ponderosae]	8.00E-60	Yes	Yes

### Identification of Putative Chemosensory-Binding Proteins

A total of 11 putative CSPs were identified from the *S. schevyrewi* antennal transcriptome. Seven of them had full-length ORFs and nine of them had the predicted signal peptide. All of them shared the typical structure of a CSP except ScosCSP3 and ScosCSP10 because these two lacked the signal peptide.

**Figure 5:**
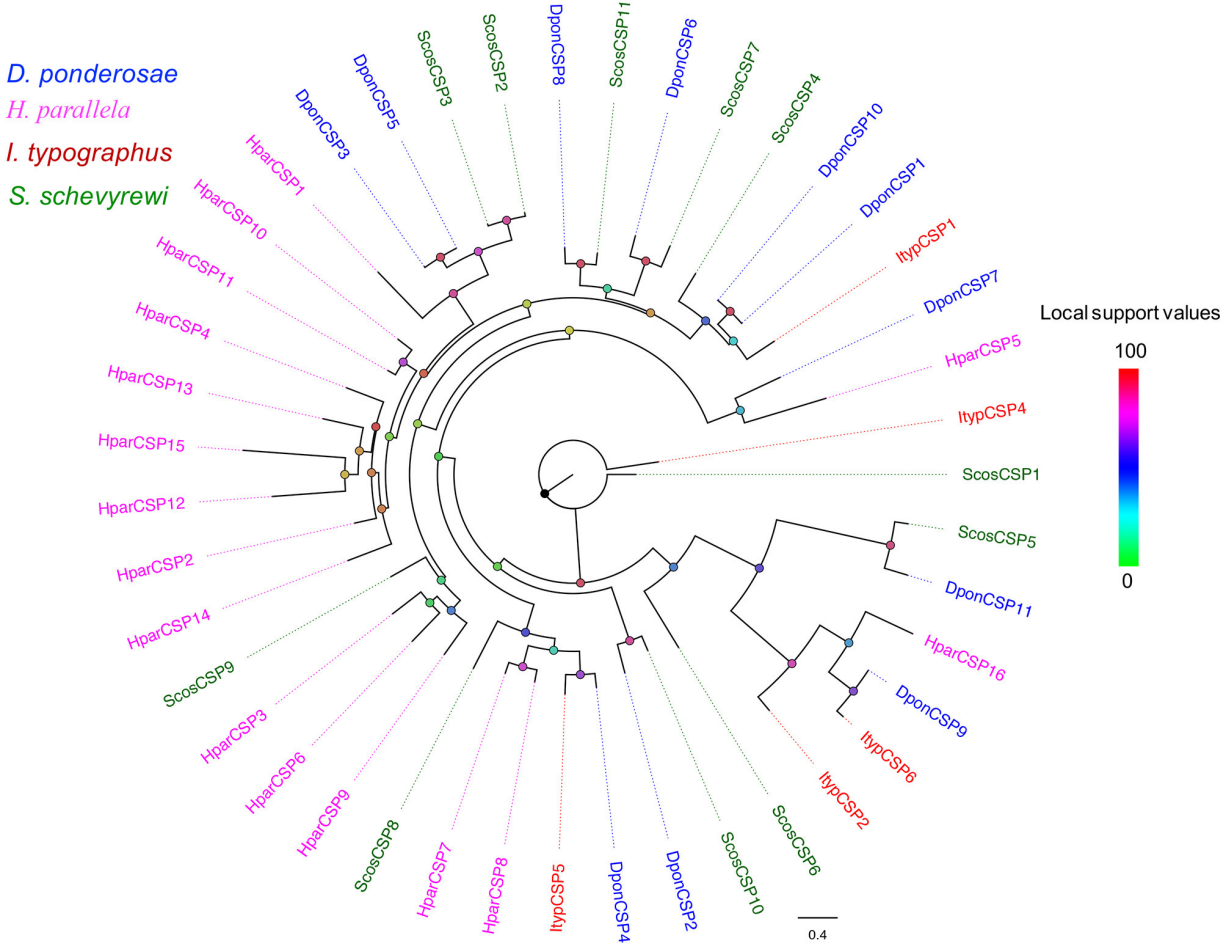
Phylogenetic tree of candidate ScosCSPs with known Coleoptera chemosensory proteins sequences. Tcas, *T. castaneum*; Dpon, *D. ponderosae*; Hpar, *H. parallela*; Ityp, *I. typographus*; Scos, *S. schevyrewi*.

All of the predicted CSPs shared relatively high identity (57–100%) with known insect CSPs. The phylogenetic analysis of the CSPs in different beetles showed that most of the ScosCSPs were clustered with orthologs of *D. ponderosae, I. typographus*, and *H. parallela* in a separate clade (Figure 5). Only ScosCSP2 and ScosCSP3 formed a small subgroup.

**Figure 6:**
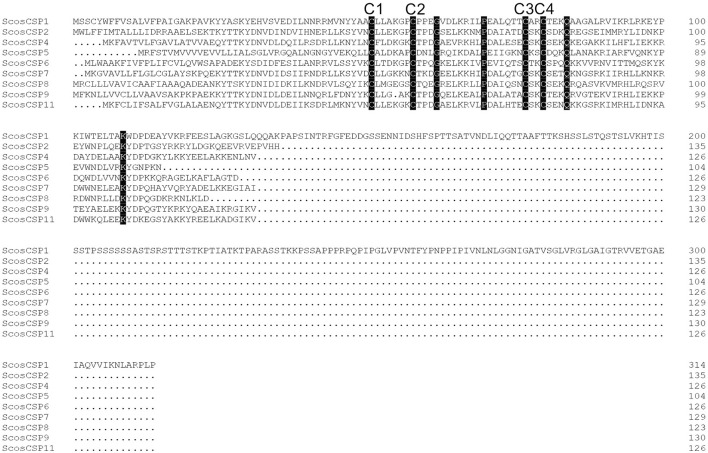
Sequence alignment of candidate ScosCSPs. The four conserved cysteine residues are marked as C1–C4. Additional residues that conserved in this species were also marked.

Information on sequence alignment, unigene reference, length, and best blastx hit of all 11 CSPs are shown in Figure 6 and Table 4.

**Table 4:** Unigene of candidate chemosensory binding proteins.

**Unigene reference**	**Gene name**	**Length (bp)**	**ORF(aa)**	**Blastx best hit**	***E*-value**	**Full length**	**Signal peptide**
CL2121.Contig1_All	ScosCSP1	948	315	XP_008193776.1 PREDICTED: chemosensory protein 6 isoform X1 [Tribolium castaneum]	3.00E-51	No	Yes
CL3677.Contig1_All	ScosCSP2	408	135	AFI45003.1 chemosensory protein [Dendroctonus ponderosae]	6.00E-65	Yes	Yes
CL3677.Contig2_All	ScosCSP3	207	68	ALR72526.1 chemosensory protein 12 [Colaphellus bowringi]	3.00E-35	No	No
Unigene2372_All	ScosCSP4	381	126	AHE13802.1 chemosensory protein 6 [Lissorhoptrus oryzophilus]	1.00E-45	Yes	Yes
Unigene3205_All	ScosCSP5	315	104	AGI05163.1 chemosensory protein 11 [Dendroctonus ponderosae]	4.00E-46	No	Yes
Unigene3958_All	ScosCSP6	381	126	AGZ04932.1 chemosensory protein 4 [Laodelphax striatella]	3.00E-86	Yes	Yes
Unigene4248_All	ScosCSP7	390	180	AGI05162.1 chemosensory protein 6 [Dendroctonus ponderosae]	1.00E-58	Yes	Yes
Unigene5446_All	ScosCSP8	372	123	AGZ04930.1 chemosensory protein 2 [Laodelphax striatella]	5.00E-56	Yes	Yes
Unigene5467_All	ScosCSP9	393	130	AGZ04940.1 chemosensory protein 12 [Laodelphax striatella]	7.00E-78	Yes	Yes
Unigene5845_All	ScosCSP10	333	110	AGI05172.1 chemosensory protein 2 [Dendroctonus ponderosae]	1.00E-52	No	No
Unigene12055_All	ScosCSP11	381	126	AHE13803.1 chemosensory protein 8 [Lissorhoptrus oryzophilus]	9.00E-67	Yes	Yes

### Tissue- and Sex-Specific Expression of Candidate ScosOBP and ScosCSP Genes

**Figure 7:**
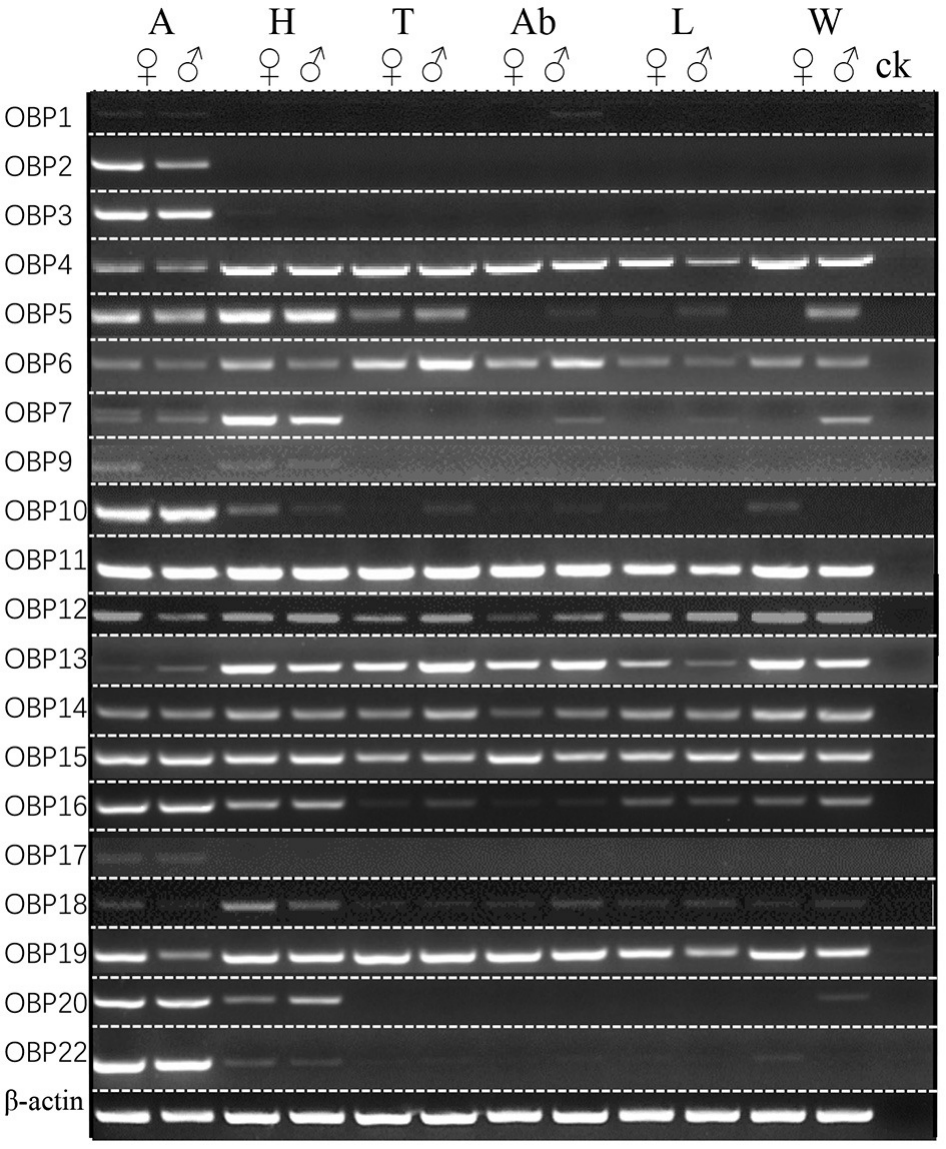
Sex- and tissue-specific expression of candidate ScosOBPs. A, antennae; H, head; T, thorax; Ab, abdomen; L, leg; W, wing; ck, control [ultra-pure water (germ-free)].

**Figure 8:**
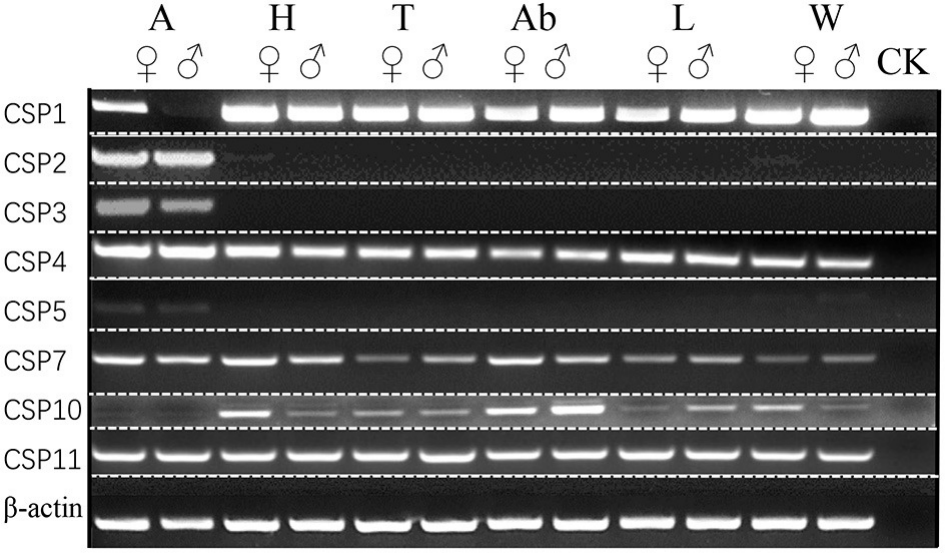
Sex- and tissue-specific expression of candidate ScosCSPs. A, antennae; H, head; T, thorax; Ab, abdomen; L, leg; W, wing; ck, control [ultra-pure water (germ-free)].

The expression patterns of ScosOBPs and ScosCSPs were analyzed by RT-PCR and are shown in Figures 7, 8. ScosOBP1, 2, 3, 7, 9, 10, 16, 17, 18, 20, and 22 were highly expressed or specifically expressed in the antennae and head tissues. Among them, ScosOBP2 and OBP18 expressed at higher levels in female antennae than in male antennae. ScosOBP4, 5, 6, 11, 12, 13, 15, 16, and 19 were generally expressed in multiple tissues. Among them, ScosOBP12 and ScosOBP19 expressions were stronger in the female than in the male antennae. ScosOBP8 and ScosOBP21 were not detected by RT-PCR possibly because their expression levels were too low to detect.

ScosCSP2, ScosCSP3, and ScosCSP5 were specifically expressed in the male and female antennae. Other ScosCSPs were expressed in multiple tissues. Among them, ScosCSP1 was not detected in male antennae and ScosCSP10 was not detected in the antennae of both the sexes. Potentially due to undetectable expression levels, ScosCSP6, ScosCSP8, and ScosCSP9 were not detected by RT-PCR.

## Discussion

The genes reported in our study provide additional knowledge on the pool of identified olfactory genes in coleopterans. Compared with a large number of studies on Lepidopteran species, the current understanding of olfactory genes in Coleoptera is mainly sourced from a few reported studies on *T. castaneum* (Engsontia et al., 2008), *Megacyllene caryae* (Mitchell et al., 2012), *I. typographus*, and *D. ponderosae* (Andersson et al., 2013), *Leptinotarsa decemlineata* (Liu et al., 2015), *H. parallela* (Yi et al., 2018), *Rhynchophorus ferrugineus* (Antony et al., 2016; Gonzalez et al., 2021), etc. *S. schevyrewi* belongs to the genus of bark beetles and shares similar biology with the related species that are destructive forest pests, such as *I. typographus* and *D. ponderosae*. Aggregation behaviors are critical for bark beetle survival and rely on chemical communication (Byers, 1989). The genes we identified might contribute to aggregation behavior and provide molecular targets for novel pest management techniques.

We identified a total of 47 OR genes in the *S. schevyrewi* antennae transcriptome. In another coleopteran, 265 candidate OR genes were annotated in the *T. castaneum* genome (Richards, 2008), which is much more than the known number of OR genes reported by other beetles. The numbers of ORs in *M. caryae* (Mitchell et al., 2012), *I. typographus* (Andersson et al., 2013), *D. ponderosae* (Andersson et al., 2013), and *H. parallela* (Yi et al., 2018) range from 43 to 57. The number of ScosORs identified in this study is consistent with that identified in these reports. Most of the predicted ORs in *S. schevyrewi* share greater identity with ORs of *D. ponderosae*, another bark beetle, indicating that these two species may be able to share the same ecological environments and detect similar semiochemicals. Functional studies in OR from bark beetles were relatively limited to only seven ORs (Hou et al., 2021; Yuvaraj et al., 2021). ItypOR46 and ItypOR49 were responsive to single enantiomers of the common bark beetle pheromone compounds ipsenol and ipsdienol, respectively (Yuvaraj et al., 2021). The other five ItypORs were responsive to monoterpenoids of different ecological origins (Hou et al., 2021). Future studies should be focused on deorphinized ScosORs with similar functions to provide potential molecular targets for detection and control methods.

We identified, in total, 22 IR genes in the *S. schevyrewi* antennae transcriptome. ScosIR8a and ScosIR25a were identified as coreceptors. The numbers of IR genes in *I. typographus, D. ponderosae*, and *H. parallela* (Yi et al., 2018) are 7, 15, and 27, respectively (Andersson et al., 2013; Yi et al., 2018). The number of ScosIRs identified in this study is considerable compared with the numbers reported in the previous studies. More than half of the predicted IRs shared relatively low identity with other coleopteran IRs. These IRs with low identity were probably not conserved in Coleoptera, and they might serve diverse functions in *S. schevyrewi*.

Within the *S. schevyrewi* antennae transcriptome, a total of 22 OBPs were predicted. Genome annotation indicated that 46 OBPs were identified in *T. castaneum* (Richards, 2008). Fewer OBPs were found in other coleopteran antennae transcriptomes. In *D. ponderosae, I. typographus*, and *H. parallela*, respectively, 31, 15, and 25 OBPs were annotated (Andersson et al., 2013; Ju et al., 2014). Our number of ScosOBPs is consistent with the numbers stated in these reports.

A total of 11 CSPs were identified in the *S. schevyrewi* antennae transcriptome. In the *T. castaneum* genome, a total of 40 CSPs were identified (Richards, 2008). Other coleopterans have fewer CSPs in their antennae transcriptomes; in *D. ponderosae, I. typographus*, and *H. parallela*, 11, 6, and 16 were annotated, respectively (Andersson et al., 2013; Ju et al., 2014). Our recorded number of ScosCSPs is comparable with these reports. The high level of sequence conservation (57–100%) indicates the function of CSPs is likely conserved among coleopterans.

All ScosOBPs and ScosCSPs contain a typical OBP and CSP motif, respectively. ScosOBP1, 2, 3, 10, 12, 14, 16, 17, 18, and 22 generally possessed the “classic” OBP motif C_1_-X_22−44_-C_2_-X_3_-C_3_-X_21−42_-C_4_-X_8−12_-C_5_-X_8_-C_6_ of coleopteran insects (Xu et al., 2009). ScosOBP4, 5, 8, 9, 11, 13, 15, and 19 generally contained the “minus-C” OBP motif C_1_-X_30−32_-C_2_-X_38_-C_3_-X_16−18_-C_4_ (Ju et al., 2014). The cysteine-spacing pattern of ScosOBP20 followed the “plus-C” OBP motif C_1_-X_24−28_-C_2_-X_3_-C_3_-X_43_-C_4_-X_13−15_-C_4a_-X_9_-C_5_-X_8_-C_6p_-X_9_-C_6a_ from *D. melanogaster* and *Anopheles gambiae* (Zhou et al., 2008). All the CSPs were conserved in having the motif C_1_-X_6_-C_2_-X_18_-C_3_-X_2_-C_4_ (Xu et al., 2009).

Scolytinae beetles respond to volatiles that emanate from both the host and non-host plants (Zhang and Schlyter, 2004; Erbilgin et al., 2007; Andersson et al., 2010). However, most individuals locate target trees by relying on an important signal called an aggregation pheromone released by beetles that have already attacked a tree (Andersson et al., 2013). Thus, olfactory signals and proteins serve critical roles in insect behavior. In this study, ScosOBP1, 2, 3, 7, 9, 10, 16, 17, 18, 20, and 22 might be important in odor perception because they were only expressed in the antennae and head, especially, ScosOBP2 and ScosOBP18. These two may be the key proteins in female olfactory behavior based on the specificity of protein expression we observed. ScosCSP2, ScosCSP3, and ScosCSP5 might also be important in olfaction due to their antennae-specific expression. Other ScosOBPs and ScosCSPs might not be involved in odor reception. Studies have shown a multitude of other roles that insect OBPs and CSPs have in Pelosi et al. (2018) releasing semiochemicals in pheromone glands (Benton, 2007), regeneration and development (Cheng et al., 2015), anti-inflammatory action (Isawa et al., 2002), nutrition (Zhu et al., 2016b), carrying visual pigments (Wang et al., 2007), and insecticide resistance (Bautista et al., 2015).

## Data Availability Statement

The datasets presented in this study can be found in online repositories. The names of the repository/repositories and accession number(s) can be found at: https://www.ncbi.nlm.nih.gov/bioproject/PRJNA732801.

## Author Contributions

XZ and WL designed the research, analyzed the data, and wrote the paper. YL gave a lot of adivces and help to revise the paper. AK, BS, and HC provided biological samples. XZ, BX, and ZQ performed the experiment. All authors contributed to the article and approved the submitted version.

## Funding

This study was funded by the National Natural Science Foundation of China (Grant No. 31660518) and the earmarked fund of the Xinjiang apricot Industrial technology (Grant No. XJCYTX-03).

## Conflict of Interest

The authors declare that the research was conducted in the absence of any commercial or financial relationships that could be construed as a potential conflict of interest.

## Publisher's Note

All claims expressed in this article are solely those of the authors and do not necessarily represent those of their affiliated organizations, or those of the publisher, the editors and the reviewers. Any product that may be evaluated in this article, or claim that may be made by its manufacturer, is not guaranteed or endorsed by the publisher.

## Supplementary Material

Supplementary Figure 1 GO classification analysis of unigenes in all-unigene. GO functions are shown in the X-axis. The right Y-axis shows the number of genes that have the GO function, and the left Y-axis shows the percentage.

Supplementary Figure 2 Uncropped gel images of candidate ScosOBPs and candidate ScosCSPs. A, antennae; H, head; T, thorax; Ab, abdomen; L, leg; W, wing; ck, control [ultra-pure water (germ free)].

Supplementary Figure 3 Uncropped gel images for candidate ScosOBPs and ScosCSPs.

Supplementary Table 1 Forward and reverse primer sequence used in RT-PCR.

Supplementary Table 2 Sequence information.

## References

[B1] Andersson M. N. Grosse-Wilde E. Keeling C. I. Bengtsson J. M. Yuen M. Li M.. (2013). Antennal transcriptome analysis of the chemosensory gene families in the tree killing bark beetles, *Ips typographus* and *Dendroctonus ponderosae* (Coleoptera: Curculionidae: Scolytinae). BMC Genom. 14:198. 10.1186/1471-2164-14-198 23517120 PMC3610139

[B2] Andersson M. N. Keeling C. Mitchell R. F. (2019). Genomic content of chemosensory genes correlates with host range in wood-boring beetles (*Dendroctonus ponderosae, Agrilus planipennis* and *Anoplophora glabripennis*). BMC Genom. 20:690. 10.1186/s12864-019-6054-x 31477011 PMC6720082

[B3] Andersson M. N. Larsson M. C. BlaŽenec M. Jakuš R. Zhang Q. H. Schlyter F. (2010). Peripheral modulation of pheromone response by inhibitory host compound in a beetle. J. Exp. Biol. 213, 3332–3339. 10.1242/jeb.044396 20833926

[B4] Angeli S. Ceron F. Scaloni A. Monti M. Monteforti G. Minnocci A.. (1999). Purification, structural characterization, cloning and immunocytochemical localization of chemoreception proteins from *Schistoceraca gregaria*. Eur. J. Biochem. 262, 745–754. 10.1046/j.1432-1327.1999.00438.x 10411636

[B5] Antony B. Soffan A. Jakse J. Mahmoud M. M. Aldosari S. A. Aldawood A. S. (2016). Identification of the genes involved in odorant reception and detection in the palm weevil Rhynchophorus ferrugineus, an important quarantine pest, by antennal transcriptome analysis. BMC Genom. 17:69. 10.1186/s12864-016-2362-6 26800671 PMC4722740

[B6] Bautista M. A. Bhandary B. Wijeratne A. J. Michel A. P. Hoy C. W. Mittapalli O. (2015). Evidence for trade-offs in detoxification and chemosensation gene signatures in *Plutella xylostella*. Pest. Manag. Sci. 71, 423–432. 10.1002/ps.3822 24796243

[B7] Benton R. (2007). Sensitivity and specificity in *Drosophila* pheromone perception. Trends.Neurosci. 30, 512–519. 10.1016/j.tins.2007.07.004 17825436

[B8] Benton R. Sachse S. Michnick S. W. Vosshall L. B. (2006). A typical memobrane topology and heteromeric function of Drosophila odorant receptors *in vivo*. PLoS. Biol. 4:e20. 10.1371/journal.pbio.0040020 16402857 PMC1334387

[B9] Benton R. Vannice K. S. Gomez-Diaz C. Vosshall L. B. (2009). Variant ionotropic glutamate receptors as chemosensory receptors in *Drosophila*. Cell. 136, 149–162. 10.1016/j.cell.2008.12.001 19135896 PMC2709536

[B10] Breugel F. V. Huda A. Dickinson M. H. (2018). Distinct activity-gated pathways mediate attraction and aversion to CO2 in *Drosophila*. Nature 564, 420–424. 10.1038/s41586-018-0732-8 30464346 PMC6314688

[B11] Briand L. Swasdipan N. Nespoulous C. Bézirard V. Blon F. Huet J. C.. (2002). Characterization of a chemosensory protein (ASP3c) from honeybee (*Apis mellifera* L.) as a brood pheromone carrier. Eur. J. Biochem. 269, 4586–4596. 10.1046/j.1432-1033.2002.03156.x 12230571

[B12] Byers J. (1989). Chemical ecology of bark beetles. Experientia. 45, 271–283.

[B13] Cao D. Liu Y. Wei J. Liao X. Walker W. B. Li J.. (2014). Identification of Candidate olfactory genes in Chilo suppressalis by antennal transcriptome analysis. Int. J. Biol. Sci. 10, 846–860. 10.7150/ijbs.9297 25076861 PMC4115196

[B14] Chen C. H. Buhl E. Xu M. Croset V. Rees J. S. Lilley K. S.. (2015). *Drosophila* ionotropic receptor 25a mediates circadian clock resetting by temperature. Nature 527, 516–520. 10.1038/nature16148 26580016

[B15] Cheng D. F. Lu Y. Y. Zeng L. Liang G. W. He X. F. (2015). Si-CSP9 regulates the integument and moulting process of larvae in the red imported fire ant, *Solenopsis invicta*. Sci. Rep. 5:9245. 10.1038/srep09245 25784646 PMC4363891

[B16] Clyne P. J. Warr C. G. Carlson J. R. (2000). Candidate taste receptors in *Drosophila*. Science 287, 1830–1834. 10.1126/science.287.5459.1830 10710312

[B17] Clyne P. J. Warr C. G. Marc R. F. Lessing D. Kim J. Y. Carlson J. R. (1999). A novel family of divergent seven-transmembrane proteins: candidate odorant receptors in *Drosophila*. Neuron 22, 327–338. 10.1016/S0896-6273(00)81093-4 10069338

[B18] Conesa A. Götz S. García-Gómez J. M. Terol J. Talón M. Robles M. (2005). Blast2GO: a universal tool for annotation, visualization and analysis in functional genomics research. Bioinformatics. 21:3674–3676. 10.1093/bioinformatics/bti610 16081474

[B19] Dobritsa A. A. van der Goes van Naters W. Warr C. G. Steinbrecht R. A. Carlson J. R. (2003). Integrating the molecular and cellular basis of odor codling in the *Drosophila* antenna. Neuron 37, 827–841. 10.1016/S0896-6273(03)00094-1 12628173

[B20] Dong X. T. Liao H. Zhu G. H. Khuhro S. A. Ye Z. F. Yan Q.. (2017). CRISPR/Cas9-mediated PBP1 and PBP3 mutagenesis induced significant reduction in electrophysiological response to sex pheromones in male *chilo suppressalis*. Insect. Sci. 26, 388–399. 10.1111/1744-7917.12544 29058383 PMC7379591

[B21] Elmore T. Ignell R. Carlson J. Smith D. (2003). Targeted mutation of a *Drosophila* odor receptor defines receptor requirement in a novel class of sensillum. J. Neurosci. 23, 9906–9912. 10.1523/JNEUROSCI.23-30-09906.2003 14586020 PMC6740877

[B22] Engsontia P. Sanderson A. P. Matthew C. Walden K. Robertson H. M. Browna S. (2008). The red flour beetle's large nose: An expanded odorant receptor gene family in *Tribolium castaneum*. Insect. Biochem. Mol. Biol. 38, 387–397. 10.1016/j.ibmb.2007.10.005 18342245

[B23] Erbilgin N. Mori R. I. Sun J. H. Stein J. D. Owen D. R. Merrill L. D. (2007). Response to host volatiles by native and introduced populations of *Dendroctonus valens* (Coleoptera: Curculionidae, Scolytinae) in North America and China. J. Chem. Ecol. 33, 131–146. 10.1007/s10886-006-9200-2 17160720

[B24] Fan L. H. Niu H. L. Zhang J. T. Liu J. L. Yang M. H. Zong S. X. (2014). Extraction and identification of aggregation pheromone components of *Scolytus schevyrewi* Semenov(Coleoptera: Scolytidae) and trapping test. Acta Ecol. Sinica 35, 892–899. 10.5846/stxb201311032656

[B25] Gomez-Diaz C. Reina J. H. Cambillau C. Benton R. (2013). Ligands for pheromone-sensing neurons are not conformatinally activated odorant binding proteins. PLoS Biology. 11:e1001546. 10.1371/journal.pbio.1001546 23637570 PMC3640100

[B26] Gonzalez F. Johny J. Walker I. I. I. W. B. Guan Q. T. Mfarrej S. Jakse J. (2021). Antennal transcriptome sequencing and identification of candidate chemoreceptor proteins from an invasive pest, the American plam weevil, *Rhynchophorus palmarus*. Sci. Rep. 11:8334. 10.1038/s41598-021-87348-y 33859212 PMC8050089

[B27] Grabherr M. C. Haas B. J. Yassour M. Levin J. Z. Tompson D. A. Amit I.. (2011). Full-length transcriptome assembly from RNA-Seq data without a reference genome. Nat. Biothchnol. 29, 644–652. 10.1038/nbt.1883 21572440 PMC3571712

[B28] Hallem E. A. Ho M. G. Carlson J. R. (2004). The molecular basis of odor coding in the Drosophila antenna. Cell 117, 965–979. 10.1016/j.cell.2004.05.012 15210116

[B29] Hanson B. S. (1999). Insect Olfaction. New York, NY: Springer.

[B30] Honson N. Gong Y. Plettner E. (2015). “Structure and function of insect odorant and pheromone-binding proteins (OBPs and PBPs) and chemosensory-specific proteins (CSPs),” in Chemical Ecology and Phytochemistry of Forest Ecosystems, ed J. T. Romeo (Oxford: Elsevier Ltd.), 227–268. 10.1016/S0079-9920(05)80010-3

[B31] Hou X. Q. Yuvaraj K. J. Roberts R. E. Zhang D. D. Uneilius C. R. Löfstedt C.. (2021). Functional evolution of a bark beetle odorant receptor clade detecting monoterpenoids of different ecological origins. Mol. Biol. Evol. msab218. 10.1093/molbev/msab218 34293158 PMC8557457

[B32] Isawa H. Yuda M. Orito Y. Chinzei Y. (2002). A mosquito salivary protein inhibits activation of the plasma contact system by binding to factor XII and high molecular weight kininogen. J. Biol. Chem. 277, 27651–27658. 10.1074/jbc.m203505200 12011093

[B33] Jacquin-Joly E. Vogt R. G. François M. C. Nagnan-Le Meillour P. (2001). Functional and expression pattern analysis of chemosensory proteins expressed in antennae and pheromonal gland of *Mamestra brassicae*. Chem. Senses 26, 833–844. 10.1093/chemse/26.7.833 11555479

[B34] Jing D. Zhang T. Bai S. Prabu S. He K. Dewer Y.. (2019). GOBP1 plays a key role in sex pheromones and plant volatiles recognition in yellow peach moth, conogethes punctiferalis (Lepidoptera: Crambidae). Insects 10:302. 10.3390/insects10090302 31533342 PMC6780721

[B35] Ju Q. Li X. Jiang J. X. Qu M. J. Guo X. Q. Han Z. J.. (2014). Transcriptome ranscriptome and tissue-specific expression analysis of obp and csp genes in the dark black chafer. Arch. Insect. Biochem. 87, 177–200. 10.1002/arch.21188 25099623

[B36] Kim M. S. Repp A. Smith D. P. (1998). LUSH odorant binding protein mediates chemosensory responses to alcohols in *Drosophila melanogaster*. Genetics 150, 711–721. 10.1093/genetics/150.2.711 9755202 PMC1460366

[B37] Knecht Z. A. Silbering A. F. Ni L. N. Klein M. Budelli G. Bell R.. (2016). Distinct combinations of variant ionotropic glutamate receptors mediate thermosensation and hygrosensation in *Drosophila*. Elife 5:e17879. 10.1101/056267 27656904 PMC5052030

[B38] Larsson M. C. Domingos A. I. Jones W. D. Chiappe M. E. Amrein H. Vosshall L. B. (2004). Or83b encodes a broadly expressed odorant receptor essential for *Drosophila* olfaction. Neuron 43, 703–714. 10.1016/j.neuron.2004.08.019 15339651

[B39] Leal W. S. (2013). Odorant reception in insects: roles of receptors, binding proteins, and degrading enzymes. Annu. Rev. Entomol. 58, 373–391. 10.1146/annurev-ento-120811-153635 23020622

[B40] Li F. Dewer Y. Li D. Qu C. Luo C. (2021). Functional and evolutionary characterization of chemosensory protein CSP2 in the whitefly, Bemisia tabaci. Pest. Manag. Sci. 77, 378–388. 10.1002/ps.6027 32741104

[B41] Li J. L. Zhang T. Li X. T. Ma S. K. (1995). Studies on the bionomics of *Scolytus seulensis* Murayama and its control. Plant Protection 21, 8–10. 10.1016/j.aspen.2008.04.001

[B42] Liu S. J. Liu N. Y. He P. Li Z. Q. Dong S. L. Mu L. F. (2012). Molecular characterization, expression patterns, and ligand-binding properties of two OBP genes from *Orthaga achatina*(Butler) (Lepidoptera: Pyralidae). Arch. Insect. Biochem. 80, 123–139. 10.1002/arch.21036 22648659

[B43] Liu Y. Sun L. J. Cao D. P. Walker W. B. Zhang Y. Q. Wang G. R. (2015). Identification of candidate olfactory genes in *Leptinotarsa decemlineata* by antennal transcriptome analysis. Font. Ecol. Evol. 19:60. 10.3389/fevo.2015.00060

[B44] Mitchell R. F. Hughes D. T. Luetje C. W. Millar J. G. Soriano-Agatónc F. Hanks L. M.. (2012). Sequencing and characterizing odorant receptors of the cerambycid beetle *Megacyllene caryae*. Insect Biochem. Mol. Biol. 42, 499–505. 10.1016/j.ibmb.2012.03.007 22504490 PMC3361640

[B45] Mitchell R. F. Schneider T. M. Schwartz A. M. Andersson M. N. McKenna D. D. (2019). The diversity and evolution of odorant receptors in beetles (Coleoptera). Insect Mol. Biol. 29, 77–91. 10.1111/imb.12611 31381201

[B46] Monteforti G. Angeli S. Petacchi R. Minnocci A. (2002). Ultrastructural characterization of antennal sensilla and immunocytochemical localization of a chemosensory protein in *Carausius morosus* Brünner (Phasmida: Phasmatidae). Arthropod Struct. Dev. 30, 195–205. 10.1016/S1467-8039(01)00036-6 18088955

[B47] Ni L. Klein M. Svec K. Budelli G. Chang E. C. Ferrer A. J.. (2015). The Ionotropic Receptors IR21a and IR25a mediate cool sensing in Drosophila. Elife. 5. 10.7554/eLife.13254 27126188 PMC4851551

[B48] Pelosi P. Calvello M. Ban L. P. (2005). Diversity of odorant binding protein and chemosensory proteins in insects. Chem. Senses 30, 1291–1292. 10.1093/chemse/bjh229 15738163

[B49] Pelosi P. Iovinella I. Zhu J. Wang G. R. Dani F. R. (2018). Beyond chemoreception: diverse tasks of soluble olfactory proteins in insects. Bio. Rev. 93, 184–200. 10.1111/brv.12339 28480618

[B50] Pelosi P. Zhou J. J. Ban L. P. Calvello M. (2006). Solube proteins in insect chemical communication. Cell. Mol. Life. Sci. 63, 1658–1676. 10.1007/s00018-005-5607-0 16786224 PMC11136032

[B51] Pertea G. Huang X. Q. Liang F. Antonescu V. Razvan S. R. Svetlana K. S.. (2003). TIGR Gene Indices clustering tools (TGICL): a software system for fast clustering of large EST datasets. Bioinformatics 19, 651–652. 10.1093/bioinformatics/btg034 12651724

[B52] Richards S. (2008). The genome of the model beetle and pest *Tribolium castaneum*. Nature 452, 949–955. 10.5167/uzh-2931 18362917

[B53] Schlyter F. Birgersson G. Byers J. A. Löfqvist J. Bergström G. (1987). Field response of spruce bark beetle, Ips typographus, to aggregation pheromo candidates. J. Chem. Ecol. 13:701–716. 24302039 10.1007/BF01020153

[B54] Stengl M. (2017). Chemosensory transduction in arthropods,” in Oxford Handbooks Online. The Oxford Handbook of Invertebrate Neurobiology, ed J. H. Byrne (Oxford University Press), 1–42. 10.1093/oxfordhb/9780190456757.013.15

[B55] Stoker R. F. Lienhard M. C. Borst A Fischbach K. F. (1990). Neuronal architecture of the antennal lobe in Drosophila melanogaster. Cell. Tissue Res. 262, 9–34. 2124174 10.1007/BF00327741

[B56] Sun H. Y. Guan L. Feng H. L. Yin J. Cao Y. Z. Xi J. H.. (2014). Functional characterization of chemosensory proteins in the scarab beetle, holotrichia oblita faldermann (Coleoptera: Scarabaeida). PLoS ONE 9:e107059. 10.1371/journal.pone.0107059 25188038 PMC4154846

[B57] Vosshall L. B. Hanson B. S. (2011). A unified nomenclature system for the insect olfactory coreceptor. Chem. Senses 36, 497–498. 10.1093/chemse/bjr022 21441366

[B58] Wang T. Jiao Y. Montell C. (2007). Dissection of the pathway required for generation of vitamin A and for *Drosophila* phototransduction. J. Cell. Biol. 177, 305–316. 10.1083/jcb.200610081 17452532 PMC2064138

[B59] Wermelinger B. (2004). Ecology and management of the spruce bark beetle Ips typographus-a review of recent research. For. Ecol. Manag. 202, 67–82. 10.1016/j.foreco.2004.07.018

[B60] Wilson R. I. (2013). Early olfactory processing in Drosophila: mechanisms and principles. Annu. Rev. Neurosci. 36, 217–241. 10.1146/annurev-neuro-062111-150533 23841839 PMC3933953

[B61] Xiao S. Sun J. S. Carlson J. R. (2019). Robust olfactory responses in the absence of odorant binding proteins. Elife: e51040. 10.7554/eLife.51040 31651397 PMC6814364

[B62] Xu P. X. Atkinson R. Jones D. N. M. Smith D. P. (2005). Drosophila OBP LUSH is required for activity of pheromone-sensitive neurons. Neuron 45, 192–200. 10.1016/j.neuron.2004.12.031 15664171

[B63] Xu Y. L. He P. Zhang L. Fang S. Q. Dong S. L. Zhang Y. J.. (2009). Large-scale identification of odorant-binding proteins and chemosensory proteins from expressed sequence tags in insects. BMC. Genom. 10:632. 10.1186/1471-2164-10-632 20034407 PMC2808328

[B64] Ye Z. F. Liu X. L. Han Q. Liao H. Dong X. T. Zhu G. H.. (2017). Functional characterization of PBP1 gene in *Helicoverpa armigera* (Lepidoptera: Noctuidae). Sci. Rep. 7:8769. 10.1038/s41598-017-08769-2 28814748 PMC5559583

[B65] Yi J. K. Yang S. Wang S. Wang J. Zhang X. X. Liu Y. (2018). Identification of candidate chemosensory receptors in the antennal transcriptome of the large black chafer *Holotrichia parallela* Motschulsky (Coleoptera: Scarabaeidae). Comp. Biochem. Phys. D 28, 63–71. 10.1016/j.cbd.2018.06.005 29980137

[B66] Yuvaraj J. K. Roberts R. E. Sonntag Y. Hou X. Q. Grosse-Wilde E. Machara A.. (2021). Putative ligand binding sites of two functionally characterized bark beetle odorant receptors. BMC Biol. 19:16. 10.1186/s12915-020-00946-6 33499862 PMC7836466

[B67] Zhang J. Wang B. Dong S. L. Cao D. P. Dong J. F. Walker W. B.. (2015). Antennal transcriptome analysis and comparison of chemosensory gene families in two closely related noctuidae moths, helicoverpa armigera and H. assulta. PloS ONE 10. 10.1371/journal.pone.0117054 25659090 PMC4319919

[B68] Zhang Q. H. Schlyter F. (2004). Olfactory recognition and behavioural avoidance of angiosperm nonhost volatiles by conifer-inhabiting bark beetles. Agr. Forest. Entomol. 6, 1–19. 10.1111/j.1461-9555.2004.00202.x

[B69] Zhou J. J. He X. L. Pickett J. A. Field L. M. (2008). Identification of odorant-binding proteins of the yellow fever mosquito *Aedes aegypti*: genome annotation and comparative analyses. Insect Mol. Biol. 17, 147–163. 10.1111/j.1365-2583.2007.00789.x 18353104

[B70] Zhu G. H. Xu J. Cui Z. Dong X. T. Ye Z. F. Niu D. J.. (2016a). Functional characterization of SlitPBP3 in *Spodoptera litura* by CRISPR/Cas9 mediated genome editing. Insect Biochem. Mol. Biol. 75, 1–9. 10.1016/j.ibmb.2016.05.006 27192033

[B71] Zhu J. Iovinella I. Dani F. R. Liu Y. L. Huang L. Q. Liu Y.. (2016b). Conserved chemosensory proteins in the proboscis and eyes of Lepidoptera. Int. J. Biol. Sci. 12, 1394–1404. 10.7150/ijbs.16517 27877091 PMC5118785

