# Supplementary material for: Identification of Candidate Olfactory Genes in Scolytus schevyrewi Based on Transcriptomic Analysis
Source: Front Physiol. 2021 Oct 4;12:717698. doi: 10.3389/fphys.2021.717698 (PMC8521011; doi:10.3389/fphys.2021.717698)
Supplement: Supplementary Figure 2 — Uncropped gel images of candidate ScosOBPs and candidate ScosCSPs. A, antennae; H, head; T, thorax; Ab, abdomen; L, leg; W, wing; ck, control [ultra-pure water (germ free)]. [file Image_2.pdf]

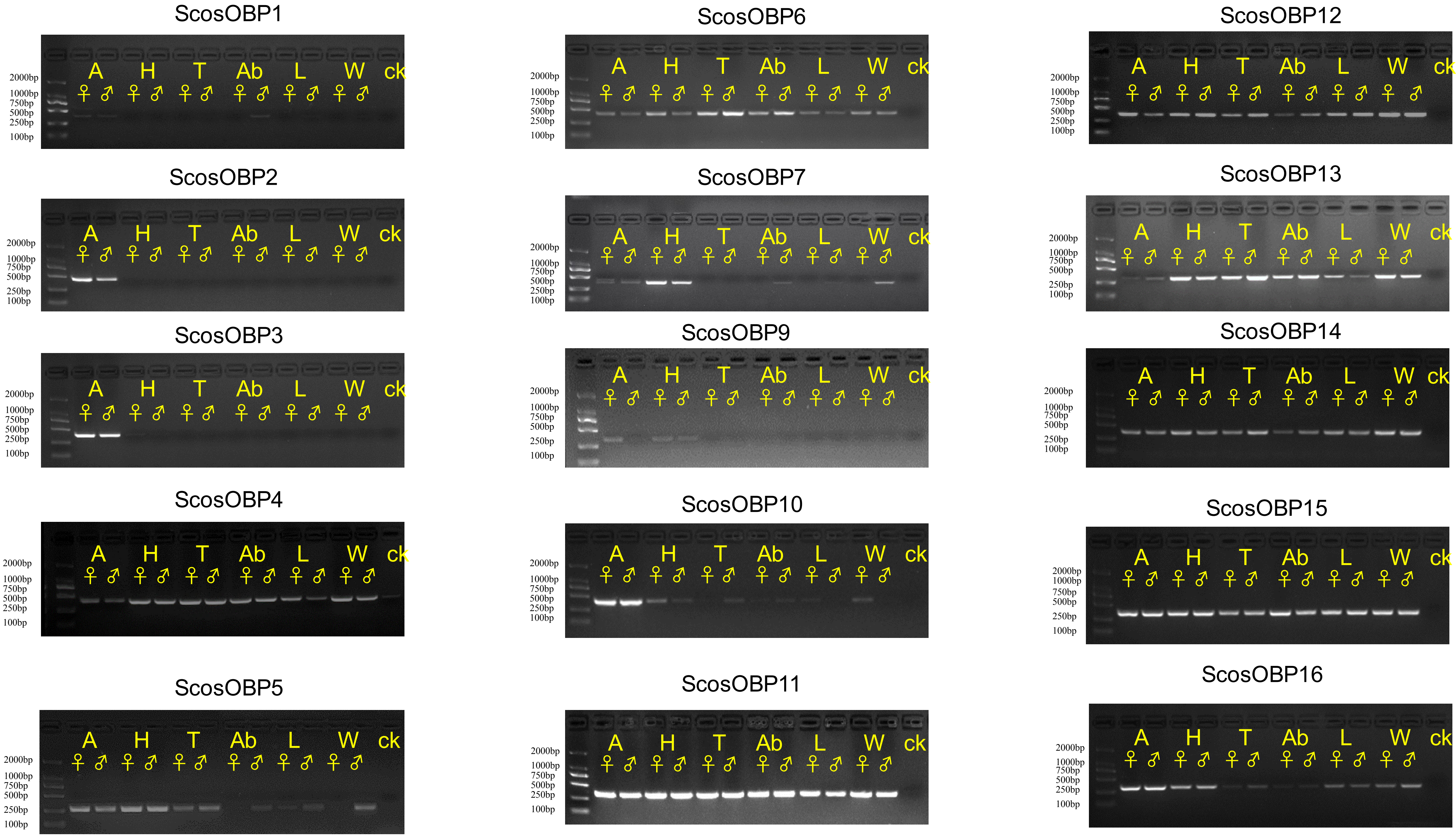
ScosOBP1
ScosOBP2
ScosOBP3
ScosOBP4
ScosOBP5
ScosOBP6
ScosOBP7
ScosOBP9
ScosOBP10
ScosOBP11
ScosOBP12
ScosOBP13
ScosOBP14
ScosOBP15
ScosOBP16
A H T Ab L W ck
♀ ♂ ♀ ♂ ♀ ♂ ♀ ♂ ♀ ♂ ♀ ♂
2000bp
1000bp
750bp
500bp
250bp
100bp
