# Supplementary material for: Identification of Candidate Olfactory Genes in Scolytus schevyrewi Based on Transcriptomic Analysis
Source: Front Physiol. 2021 Oct 4;12:717698. doi: 10.3389/fphys.2021.717698 (PMC8521011; doi:10.3389/fphys.2021.717698)
Supplement: Supplementary Figure 3 — Uncropped gel images for candidate ScosOBPs and ScosCSPs. [file Image_3.pdf]

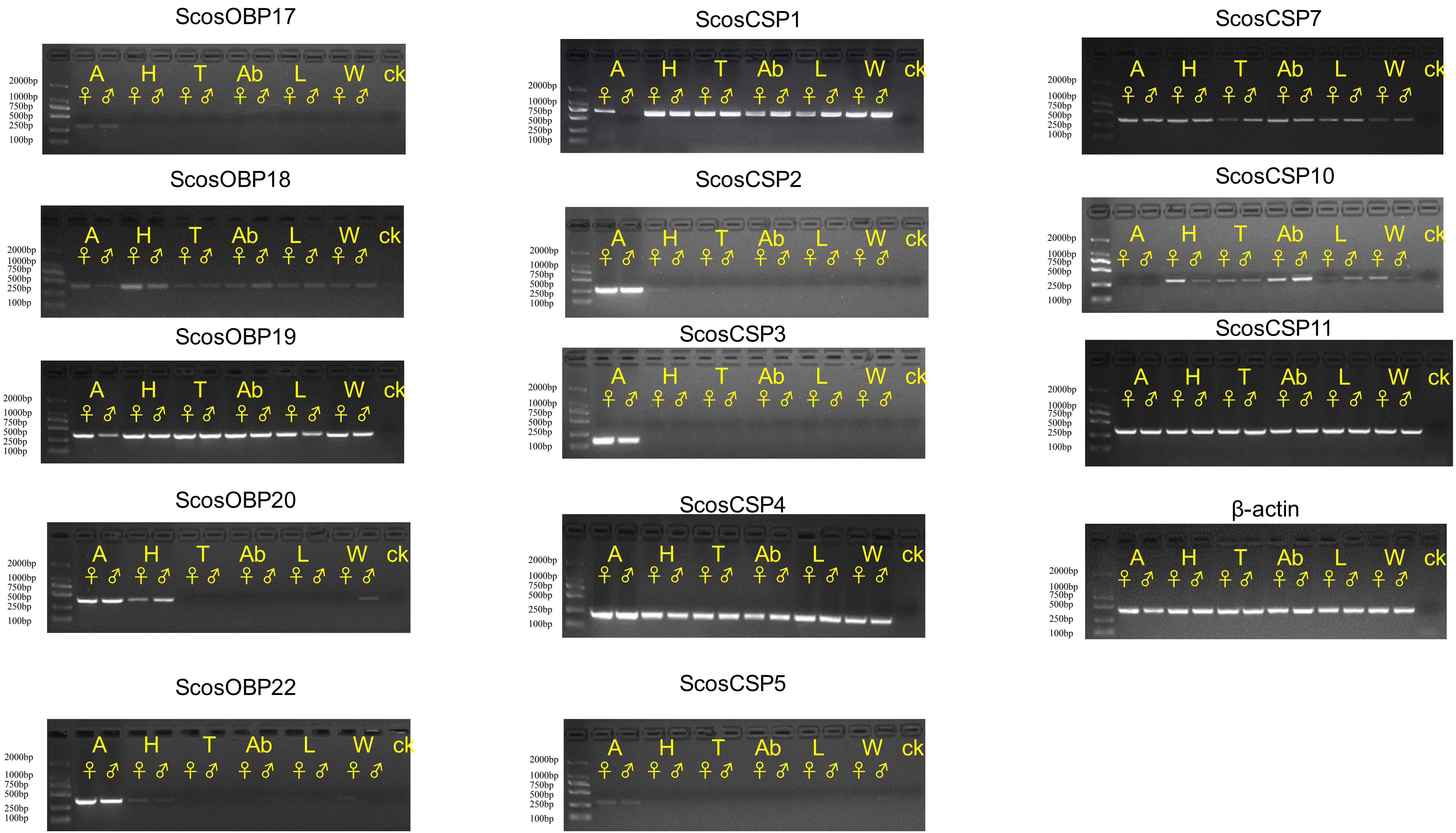
ScosOBP17
ScosOBP18
ScosOBP19
ScosOBP20
ScosOBP22
ScosCSP1
ScosCSP2
ScosCSP3
ScosCSP4
ScosCSP5
ScosCSP7
ScosCSP10
ScosCSP11
β-actin
A H T Ab L W ck
♀ ♂ ♀ ♂ ♀ ♂ ♀ ♂ ♀ ♂ ♀ ♂
2000bp
1000bp
750bp
500bp
250bp
100bp
