# Supplementary material for: Identification of Candidate Olfactory Genes in Scolytus schevyrewi Based on Transcriptomic Analysis
Source: Front Physiol. 2021 Oct 4;12:717698. doi: 10.3389/fphys.2021.717698 (PMC8521011; doi:10.3389/fphys.2021.717698)
Supplement: Supplementary Table 1 — Forward and reverse primer sequence used in RT-PCR. [file Table_1.docx]

Table S1-1 Forward and reverse primer sequence used in RT-PCR

| Unigenes | Forward primer sequence | Reverse primer sequence | Amplification length (bp) |
| --- | --- | --- | --- |
| ScosOBP1 | CTGCTAAATACTGCCGCTTGTGA | TCCTTGTTCCTCCCGAAGCACTC | 371 |
| ScosOBP2 | ATAACACGGGCTTCCTTTACAAA | TATGTGTCAACCAGGCATTATCA | 439 |
| ScosOBP3 | TTTATGTTCGGCTTGTTTGTCTT | TGTAGATGTCAGGTTGCTTTTGG | 379 |
| ScosOBP4 | CGCTGTAGTGTTCTGCTTCGTAA | GAGGAGCGGTATGCTTGAAGAAA | 395 |
| ScosOBP5 | TTTACTCTCTGACCGACCAACAA | GGTCTTGGCTTGGTCGCCTTTCA | 251 |
| ScosOBP6 | TATACAGTCGGCACCTCCAGTC | CGTTCCTCTTTGTCTAATCGTTT | 387 |
| ScosOBP7 | ATTTTCCTTCTTACGGTGGGTGT | CTCCATGGGATCGTCGAAGTCTT | 351 |
| ScosOBP8 | CGCTCGCTTTTCTGCTCGTTATC | GCCGTTTCTTTCAGGGTCGCTTT | 355 |
| ScosOBP9 | GAAGCCCTGGACAAAATAATGGA | TCTGGTAAAGACACTTGCCTGCC | 313 |
| ScosOBP10 | TGGGGCTTATTGATTTTTGCTTT | CAGGCATTTGGCTATTTCGTAAG | 381 |
| ScosOBP11 | TTTTCTTATTTTTTGTCTCCGCA | AGCCGTATCTTCTTTGGTCTGTT | 335 |
| ScosOBP12 | CTTGTCTTTGTTCTGGCGTCGTT | TTTTGTTACTGCCTGTTGCGTTG | 381 |
| ScosOBP13 | GCGAAGCAGAGCGAGAAAAAACT | CCTCGGGCGTTTCCGCTCCTTTG | 307 |
| ScosOBP14 | AAGAGCAACGGAAAAAAATCATC | CTGTTTTTCGTCCTTCTTCTCCT | 353 |
| ScosOBP15 | TTGCTAACGCCGACTTTACCGAC | CGTTTCTCGCTGCTTTCGTAGTA | 343 |
| ScosOBP16 | ATGTACGTCGTCAGAGAATTGGC | GCTCTTGGTGGTTTCCCTTAGTT | 315 |
| ScosOBP17 | ACCTTTGCTGCTGAGCTTGGC | GTTTCGAGACACTTGCGGATTTG | 297 |
| ScosOBP18 | GTCTTGACGGGCGTGACCTCT | TGCATCTTCGGAAAGCACTCG | 365 |
| ScosOBP19 | AGAACAAAGATAAAGCCGTCCTG | GATGATGATGATGATGTTCGTCG | 333 |
| ScosOBP20 | AATGGAGTGCGTCAAGGATAACC | TTTTTCAAACAGACACTGAGCGG | 378 |
| ScosOBP21 | AACACTTATCTTATGCCTCTTTG | TGTGCGTTACGGTCTACCTCCCA | 177 |
| ScosOBP22 | GTTAGTCACCGCCTCTGTTTCCT | TCCGCCTTGTACATGCAATAGTT | 357 |

Table S1-2 Forward and reverse primer sequence used in RT-PCR

| Unigenes | Forward primer sequence | Reverse primer sequence | Amplification length (bp) |
| --- | --- | --- | --- |
| ScosCSP1 | CTTCCTGCTACTGGTTCTTCGTT | TATGGTGGGTTTAGTTGATGTCG | 671 |
| ScosCSP2 | ACAACGAGCGACTGCTCAAGA | CCTCTTCTTGTTTCCCGTCCA | 264 |
| ScosCSP3 | GATTGCAGCAAGTGTAGCGA | CGATTCTTCCCGTCCAAATA | 152 |
| ScosCSP4 | AAGAACTATTTGAACTGTTTTTT | TCAGGTACTTGCCGTCGGGGTCG | 217 |
| ScosCSP5 | TGGTGGAGGTGGTTCTTTTG | AGTTCTTGGGGTTGCCGTAT | 285 |
| ScosCSP6 | TGCGTTCTCCAGGTATGGTCGGC | GGCTCTCTGCTTCTTTGGGTCGT | 303 |
| ScosCSP7 | GCCTCGCCTACTCTAAACCG | AATGGCGATTCCCTCTTTCT | 347 |
| ScosCSP8 | ATGAGGTGCCTTCTGCTGGTTGC | AAGTTCTTCCTCTTGTCTCCCTG | 359 |
| ScosCSP9 | AAGAACTTGCTCGTTGTTTGCCT | AGACCTTGATGCCTCGCTTGATG | 385 |
| ScosCSP10 | ACGACCATCTGGATATCGACACG | AAACGGACAAGAACTCTCTAAAC | 291 |
| ScosCSP11 | CTGATATTTTCTGCGCTCTTCG | GACAAAGAAGGAAGTTACGCCA | 325 |
| β-Actin | ACCACCGTTCACGCCACTAC | TTGCTGTTCAAAGCAATACCG | 392 |
