# Supplementary material for: Identification of Candidate Olfactory Genes in Scolytus schevyrewi Based on Transcriptomic Analysis
Source: Front Physiol. 2021 Oct 4;12:717698. doi: 10.3389/fphys.2021.717698 (PMC8521011; doi:10.3389/fphys.2021.717698)
Supplement: Supplementary Figure 1 — GO classification analysis of unigenes in all-unigene. GO functions are shown in the X-axis. The right Y-axis shows the number of genes that have the GO function, and the left Y-axis shows the percentage. [file Image_1.pdf]

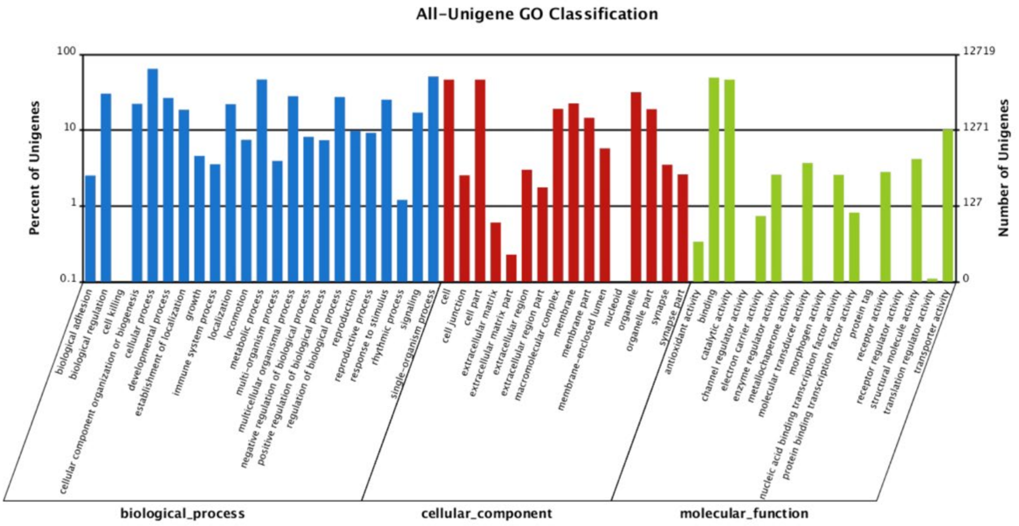

Figure S1 GO classification analysis of Unigenes in All-Unigene. GO functions is showed in X-axis. The right Y-axis shows the number of genes which have the GO function, and the left Y-axis shows the percentage.
